# Mitochondria Dictate Progressive Versus Bimodal Aging of the Mammary Gland

**DOI:** 10.1111/acel.70661

**Published:** 2026-08-09

**Authors:** Edmund Charles Jenkins, Mrittika Chattopadhyay, Thelma Mashaka, Miguel Torres‐Martin, Daniela Sia, Igor Bado, Doris Germain

**Affiliations:** ^1^ Division of Hematology/Oncology, Department of Medicine Tisch Cancer Institute, Icahn School of Medicine at Mount Sinai New York New York USA; ^2^ Center for Genomic Regulation, Barcelona Institute of Science and Technology Barcelona Spain; ^3^ Division of Liver Diseases, Department of Medicine Tisch Cancer Institute, Icahn School of Medicine at Mount Sinai New York New York USA; ^4^ Department of Oncological Sciences Tisch Cancer Institute, Icahn School of Medicine at Mount Sinai New York New York USA

**Keywords:** aging, mammary gland, mitochondria‐nuclear communication

## Abstract

The incidence of breast cancer shows its largest peak around the age of 65 but also a peak around 45 indicating a bimodal distribution. While some potential explanations, such as age‐related enrichment in specific breast cancer sub‐types and BRCA1/2 mutations were proposed, they do not explain why the peaks are at 45 and 65, raising the possibility that the way the breast ages may play a role. Here using two mouse models, we show two distinct aging patterns of the mammary gland; one being progressive, the other bimodal. In the bimodal model, waves of overlapping genes and proteins associated with breast cancer pathways are observed at 11 and 19 months but are absent at 3 and 14 months. Further, in the bimodal model, the mammary glands at 11 and 19 months are more permissive to the growth of cancer cells but not at 14 or > 22 months, while in the progressive model, the growth of cancer cells increases starting at 14 months of age. Using scRNAseq, we established a bimodal aging signature. Since 11 and 19 months in mice correlate with 45 and 65 in humans, we tested the mammary gland‐derived bimodal signature in two breast cancer databases and found that the signature is enriched in women diagnosed at 45 and 65. Therefore, our study raises the possibility that distinct patterns of aging of the breast exist and that they may contribute to the bimodal distribution of breast cancer. Further, our study adds to the growing evidence of non‐linear aging.

## Graphical abstract

Breast cancer shows a bimodal age distribution with a peak at 45 and higher peak at 65. Using mice with different mitochondrial genomes, we show progressive and bimodal patterns of aging of the mammary gland with distinct pro‐tumorigenic age windows. This finding may shed light on the bimodal distribution in humans.

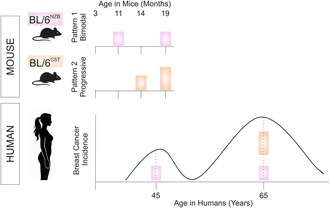

## 1 Introduction

The incidence of breast cancer increases with age and the largest peak of incidence is observed around the age of 65 (Benz 2008). However, another peak is observed at 45 indicating a bimodal age distribution (Allott et al. 2020; Anderson et al. 2006; Desai and Guddati 2022). Because breast cancers are classified based on the status of the estrogen receptor alpha (ERα) and younger women tend to develop ERα‐negative breast cancers, while breast cancers in post‐menopausal women tend to be positive for the ERα (Allott et al. 2020), one interpretation of the bimodal distribution of breast cancers is that it simply reflects ERα status. However, this interpretation does not explain the age component of this distribution and why the peaks are at 45 and 65 specifically.

Further, the early peak observed around the age of 45 is interpreted as being due to the presence of pathologic variants such as BRCA1. However, a detailed study focusing on the age at diagnosis of patients carrying any of 5 pathologic mutations (BRCA1, BRCA2, ATM, CHEK2, PALB2) found that in these patients, the age of incidence peaks between the age of 26 and 30 (Daly et al. 2023). Therefore, currently neither ERα status nor pathological mutations can explain the bimodal age distribution of breast cancer.

Mitochondrial dysfunction is a hallmark of aging (Balaban et al. 2005; Bratic and Larsson 2013; Guo et al. 2023; Harrington et al. 2023; Miwa et al. 2022). Each mitochondrion contains a small circular genome of 16 kDa, which encodes only 13 proteins, while individual mitochondrion contains over 1000 proteins. Therefore, most mitochondrial proteins are encoded by nuclear genes. The canonical function of mitochondria is the production of ATP via the activity of the electron transport chain (ETC). Some complexes of the ETC contain both mitochondrial and nuclear encoded subunits, where the mitochondrial‐encoded subunits are an essential core for the function of these complexes and consequently affect global mitochondrial fitness. Therefore, the functional integrity of mitochondria requires a unique coordination between two genomes and relies on a robust mitochondria‐nucleus communication. This inter‐organelle communication not only impacts the integrity of mitochondria but also global nuclear gene expression through the activation of the integrated stress response, which implicates several transcription factors (Anderson and Haynes 2020; Costa‐Mattioli and Walter 2020; Kenny and Germain 2017; Pakos‐Zebrucka et al. 2016) and the effects of mitochondrial metabolites that act as direct activators or repressors of epigenetic regulators (Chakrabarty and Chandel 2021; Chatterjee et al. 2022; English et al. 2020; Mohammed et al. 2020; Steinert et al. 2021; Wiese and Bannister 2020; Xu et al. 2021).

The importance of the mitochondria‐nuclear communication has been validated using cybrids, which are cells that have the same nuclear genome but carry different mitochondria genomes. Studies using cybrids have demonstrated the profound impact of mitochondrial genetics on nuclear gene expression in multiple diseases (Cruz‐Bermudez et al. 2017; Danese et al. 2022; Kaipparettu et al. 2010; Park et al. 2016; Swerdlow et al. 2017). Conversely, the same mitochondria DNA mutations or polymorphism in cells with different nuclear genomes have different impact notably on the risk of breast cancer (Bai et al. 2007; Canter et al. 2005; Covarrubias et al. 2008; Kulawiec et al. 2009). These observations suggest that each individual's mitochondria‐nucleus communication plays a role in their overall transcriptome and consequently their health and susceptibility to diseases.

In support of this possibility, the Enriquez group used the fact that mitochondrial DNA is maternally inherited to generate mice with the C57BL/6 nuclear genetic background (BL/6) but differ in mitochondrial genomes and carry either the C57BL/6 or NZB/OlaHsd (^C57^ or ^NZB^) mitochondria (Latorre‐Pellicer et al. 2016). Since the number of nucleotide differences in the mitochondrial DNA between NZB/OlaHsd and BL/6C57 mice is within the same range as that observed between haplotypes in the human population, these mice represent a model that reflects the natural diversity in mitochondrial genetics in humans (Latorre‐Pellicer et al. 2016).

The original analysis of these mice led to the discovery that the BL/6^NZB^ mice of both sexes live longer and show wide differences in nuclear gene expression in the heart and the liver. In this study however, only two time points were investigated: young (12 weeks) and old (2 years).

Using these mice, we initiated the current study to analyze the aging of the mammary gland using time points that span the decline in ovarian function over aging in female mice (Nelson et al. 1982). Surprisingly, we found that while the BL/6^C57^ females show non‐overlapping, age‐specific changes and the expected progressive involution of the mammary gland over aging, the BL/6^NZB^ females show a bimodal aging pattern. This bimodal pattern is associated with windows where the mammary glands are more permissive to the growth of cancer cells. As most biological pathways associated with aging are conserved evolutionarily (Cohen 2018), we used data generated in mice for validation in human breast cancer databases and found that the bimodal signature is enriched in breast cancers diagnosed at 45 and 65. The discovery of two distinct patterns of aging of the mammary gland suggests that the bimodal pattern may contribute to the peak at 45 and that both the progressive and bimodal patterns contribute to the larger peak at 65. Further, our study adds to the growing evidence of non‐linear aging (Fehlmann et al. 2020; Lehallier et al. 2019; Shen et al. 2024).

## 2 Methods

### 2.1 Mice

All mouse experiments were conducted per an approved protocol by the Institutional Animal Care and Use Committee (IACUC). BL/6^C57^ and BL/6^NZB^ mice were originally generated and kindly provided by Dr. José Antonio Enríquez. The mice were housed in vivariums at the Icahn School of Medicine at Mount Sinai with *ad libitum* access to food and water. BL/6^C57^ and BL/6^NZB^ female mice were aged and harvested at specified time points (*n* = 5 per timepoint for each genotype), and mammary glands were harvested and stored appropriately for subsequent analysis. The estrous cycle stage of all female mice was determined prior to experimentation. To collect vaginal epithelial cells, approximately 50 μL of phosphate‐buffered saline (PBS) was aspirated into a pipette tip and gently inserted into the vaginal opening. The PBS was flushed in and out of the vaginal canal two to three times to collect cells. The resulting cell suspension was then evenly distributed onto three microscope slides, forming a thin smear, and left to air dry. Slides were subsequently stained using Modified Giemsa Stain (Sigma‐Aldrich, GS500‐500 mL) for 2–3 min, followed by a rinse with water. The slides were examined under a microscope to determine the estrous cycle stage as described (McLean et al. 2012; Chattopadhyay et al. 2025).

The E0771 murine mammary carcinoma cell line, originally derived from a spontaneous mammary tumor in C57BL/6 mice, was used to establish orthotopic tumors in the mammary glands of mice, following a previously published protocol with minor modifications (Chattopadhyay et al. 2025). A total of 1 × 10^5^ E0771 cells, suspended in a 1:1 mixture of Hank's Balanced Salt Solution (HBSS) and growth factor‐reduced Matrigel (GIBCO) in a final volume of 50 μL, were injected into the bilateral inguinal mammary glands of estrus‐stage‐matched (Estrus phase) virgin female mice from two strains: BL/6^C57^ and BL/6^NZB^. Injections were performed at 3, 6, 11, and 19 months of age. Tumor growth was monitored three times per week using electronic calipers, measuring the longest (length) and widest (width) tumor diameters. Tumor volume was calculated using the modified ellipsoid formula: volume = ½ × (length × width^2^) as described previously (Chattopadhyay et al. 2025).

For measurement of Estradiol levels in mice serum, blood was collected from estrus cycle matched (Estrus phase) virgin mice at different ages. Blood from pregnant mice (late stage) was used a positive control. Serum was separated from all samples and estradiol levels were measured using the Elisa Kit from Enzo (ADI‐900‐174).

### 2.2 Wholemounts

Mouse abdominal mammary glands were harvested, mounted on microscope slides, and air‐dried briefly. The glands were then fixed in Carnoy's fixative (75% Ethanol, 25% glacial acetic acid) overnight. Subsequently, they were washed in 70% ethanol for 15 min, followed by a 5‐min water rinse, and stained overnight in carmine alum solution (1 g carmine (Sigma C1022) and 2.5 g aluminum potassium sulfate (Sigma A7167) in 500 mL dH_2_O) according to standard protocol. After staining, the carmine‐stained whole mounts were rinsed in MilliQ‐filtered water for 5 min and dehydrated through an alcohol series (70%, 95%, 100% EtOH, 15 min for each step). The glands were cleared in xylene and mounted using a Permount mounting medium. Finally, the whole mounts were imaged using a Zeiss Stemi 300 microscope.

### 2.3 Mammary Branching Assessment

To quantify branching in mouse mammary gland whole‐mount images, we developed an image processing pipeline using ImageJ/FIJI (v1.54f). GRB images were opened and split into individual channels to isolate the channel containing the highest contrast between duct and surrounding tissue. Background subtraction was performed by subtracting one channel from another, followed by normalization through division by the red channel twice, both in 32‐bit format, to correct for uneven staining. Shading correction was applied using a second‐degree polynomial fit in both x and y directions with a regularization parameter of 2 to address illumination artifacts. The resulting image was converted to 8‐bit, and contrast was enhanced with a saturation limit of 0.35%.

For segmentation, the Yen thresholding method was applied to generate a binary mask, with the background set to black. Morphological operations were used to refine the mask: dilation (twice) to fill gaps, de‐speckling (four times) to remove noise, and erosion (twice) to restore structure boundaries. The binary image was skeletonized to reduce mammary gland structures to single‐pixel‐wide centerlines. Branching morphology was quantified using the “Analyze Skeleton (2D/3D)” plugin, with no pruning, to measure branch number, branch lengths, junction points, and endpoints. Results were saved as TIFF images and quantitative data tables. The pipeline was automated using an ImageJ macro to ensure reproducibility across samples.

### 2.4 H&E, Masson Trichome Stain and Immunohistochemistry

Mammary glands were fixed in 10% formalin and subsequently processed and paraffin‐embedded for sectioning by the Mt. Sinai Biorepository Core Facility. Formalin Fixed Paraffin Embedded (FFPE) sections were prepared and stained for hematoxylin and eosin (H&E) according to standard protocol. Additional unstained slides were processed for collagen using the Fisher Scientific Epredia Richard‐Allan Scientific Masson Trichrome Kit following the manufacturer's instructions. Stained slides were imaged using a Zeiss AX10 Microscope. Quantification of collagen content was performed by calculating the percentage of stromal collagen per mammary gland and assessing periductal thickness using an arbitrary scoring system ranging from 1 (thin/filamentous collagen) to 5 (thick collagen). This scoring was conducted by a blinded observer.

For immunohistochemical analysis, tissue sections were first deparaffinized by immersing them in xylene twice for 5 min each. This was followed by gradual rehydration through a descending ethanol gradient (100%, 90%, and 70%), ending with distilled water. Antigen retrieval was performed by incubating the sections in 10 mM sodium citrate buffer (pH 6.0) at 90°C–100°C for 30 min. Slides were then allowed to cool gradually to room temperature and rinsed twice with Tris‐buffered saline (TBS). Immunostaining was carried out following the manufacturer's protocol for the ImmPRESS Excel Amplified Polymer Kit (Vector Laboratories, Cat# MP‐7601 and MP‐7602). Primary antibodies against Vitronectin (Abcam, Cat# AB235987) and ITGA11 (Abcam, Cat# AB316249) were applied at a 1:100 dilution and incubated overnight at room temperature in a humidified chamber.

### 2.5 Mammary Gland Decellularization

Freshly dissected mammary glands were decellularized by immersion in 1% SDS in TBS containing penicillin/streptomycin and DNase (1 U/mL) in a 15 mL Falcon tube. The tubes were shaken at room temperature for 48 h with 6 complete changes of the decellularization solution. Subsequently, the glands underwent an additional 48‐h period with 6 complete changes of water. After decellularization, the mammary glands were frozen in 10% DMSO in TBS to preserve the tissue integrity before the samples were sent for proteomics analysis.

### 2.6 Proteomics

#### 2.6.1 Protein Digestion

Decellularized mammary glands were resuspended in 50 μL of 8 M Urea, 50 mM EPPS and treated with DL‐dithiothreitol (5 mM final concentration) for 2 h at 37°C with shaking (1400 rpm) on a Thermomixer (Thermo Fisher). Free cysteine residues were alkylated with 2‐iodoacetamide (10 mM final concentration) for 30 min at 25°C in the dark. Urea was diluted to 2 M with Ammonium Bicarbonate (ABC) 100 mM and PGNase added for 2 h 37°C with shaking (1400 rpm). LysC (1:100 enzyme: protein) was added, followed by incubation for 2 h at 37°C at 1400 rpm. Finally, trypsin (1:100 enzyme: protein) was added, followed by overnight incubation at 37°C at 1400 rpm. After the overnight incubation, the digest was acidified to pH < 3 with the addition of 50% trifluoroacetic acid (TFA), and the peptides were desalted on 3‐plug C18 (3 M Empore high performance extraction disks) stage tips. Briefly, the stage tips were conditioned by sequential addition of (i) 100 μL Methanol, (ii) 100 μL 70% Acetonitrile (ACN)/0.1% TFA, (iii) 100 μL 0.1% TFA twice. Following conditioning, the acidified peptide digest was loaded onto the stage tip. The stationary phase was washed once with 100 μL of 0.1% Formic Acid (FA). Finally, samples were eluted using 50 μL of 70% ACN/0.1% FA twice. Eluted peptides were dried under vacuum followed by reconstitution in 12 μL of 0.1% FA, sonication and transfer to an autosampler vial. Peptide yield was quantified by NanoDrop (Biotek Synergy H1).

#### 2.6.2 MS Analyses

Peptides were separated on a 50 cm column composed of C18 stationary phase (Thermo Fisher ES903) using a gradient from 0.5% to 25% buffer B over 100 min, to 50% over 15 min, to 90% in 5 min (buffer A: 0.1% FA in HPLC grade water; buffer B: 99.9% ACN, 0.1% FA) with a flow rate of 300 nL/min using a nanoAcquity Hplc system (Waters). MS data were acquired on a Eclipse mass spectrometer (Thermo Fisher Scientific) using a data‐independent acquisition (DIA) method. The method consisted of one MS1 scan, 400,000 AGC target, maximum injection time of 50 msec, scan range of 380–985 m/z and a resolution of 120 K. Fragment ions were analyzed in 60 DIA windows, maximum injection time of 40 msec, 100,000 AGC target at a resolution of 15 K.

#### 2.6.3 DIA Data Analysis

Raw data files were processed using Spectronaut version 18.5 (Biognosys) and searched with the PULSAR search engine with a mouse UniProt protein database downloaded on 2022/09/26 (94,765 entries). Cysteine carbamidomethylation was specified as fixed modifications, while Methionine oxidation, acetylation of the protein N‐terminus and Deamidation (NQ) were set as variable modification. A maximum of two trypsin missed cleavages were permitted. Searches used a reversed sequence decoy strategy to control peptide false discovery rate (FDR) and 1% FDR was set as threshold for identification. Unpaired *t*‐test was used to calculate *p*‐value in differential analysis; volcano plot was generated based on log2FC and *q*‐value (multiple testing corrected *p*‐value using Benjamini‐Hochberg method). A *q*‐value of ≤ 0.05 was considered the statistically significant cut‐off.

### 2.7 Bulk RNA Sequencing

Mammary glands of BL/6^C57^ and BL/6^NZB^ females at 3, 11, 14 and 16 months of age were flash frozen on liquid nitrogen and sent to Azenta for bulk RNA sequencing. The RNA sequencing data were analyzed using BioJupies, an interactive online platform that automates the generation of principal component analysis, hierarchical clustering, and differential gene expression analysis. Gene Set Enrichment Analysis was performed using Enrichr, focusing on the top differentially expressed genes and identifying enriched biological pathways associated with the dataset.

**FIGURE 1:**
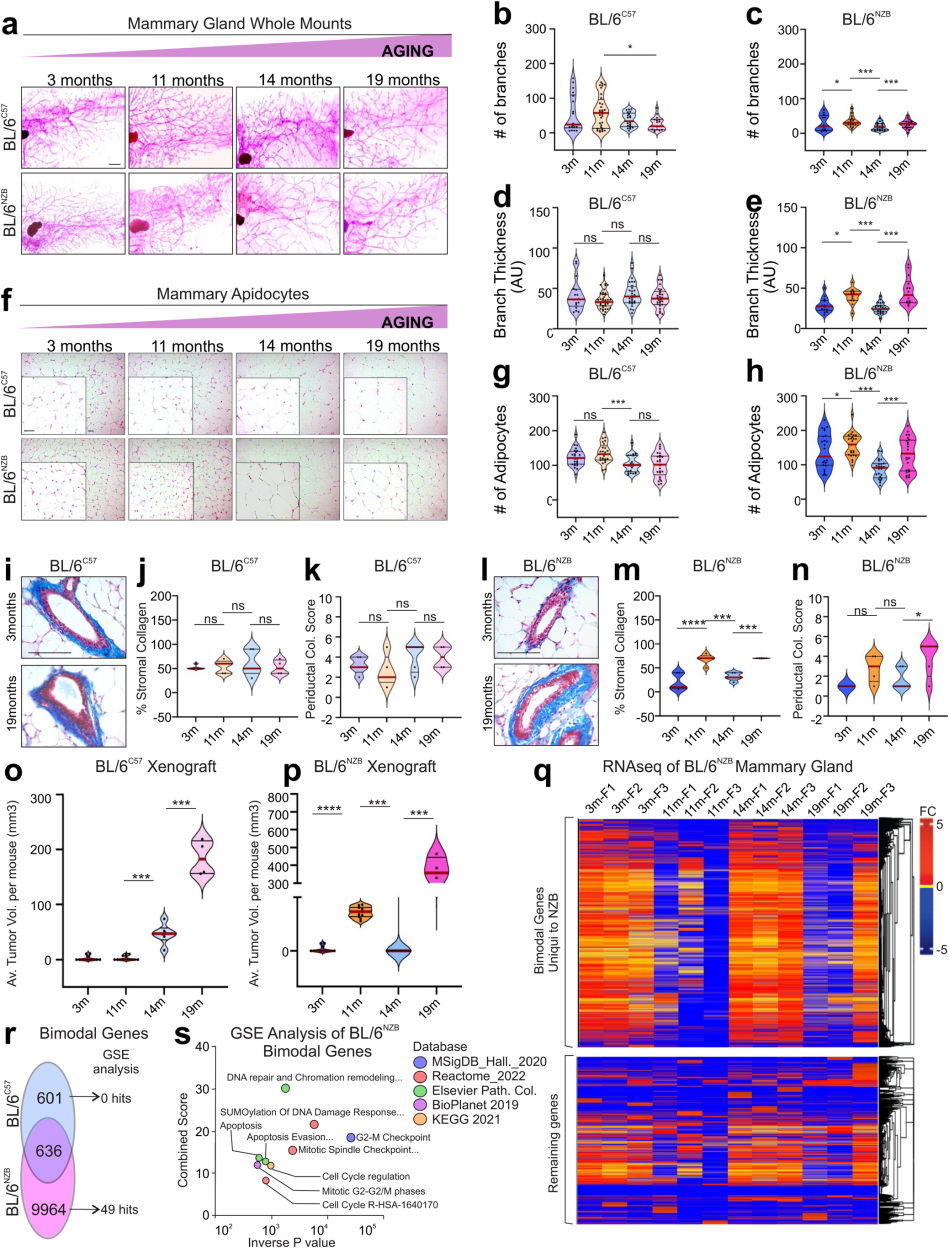
Mammary glands show non‐linear aging in BL/6^NZB^ mice. (a) Mammary gland whole mounts of BL/6^C57^ or BL/6^NZB^ mice at 3, 11 14, or 19 months of age. (b, c) Quantification of the number of branch points (> 3) in mammary gland whole mounts as in (a). *n* = 8 mice at 3 months and *n* = 5 at other time points and dots indicate 5 area per mouse. (d, e) Quantification branch thickness (Arbitrary Units) in mammary gland whole mounts as in (a). *n* = 5 mice per time point and dots indicate 5 area per mouse. (f) Staining of mammary adipocytes of BL/6^C57^ or BL/6^NZB^ mice at 3, 11, 14, or 19 months of age. (g, h) Quantification of the number of adipocytes per field, *n* = 5 mice per time point and dots indicate 5 area per mouse. Masson's Trichrome Staining (MTS) for collagen of mammary gland sections from BL/6^C57^ (i) or BL/6^NZB^ (l) mice. Quantification of stromal collagen in mammary glands from BL/6^C57^ (j) or BL/6^NZB^ (m) mice at 3, 11, 14, and 19 months of age. *n* = 4 biological replicates. Periductal collagen scoring of mammary glands from BL/6^C57^ (k) or BL/6^NZB^ (n) mice at 3, 11, 14, and 19 months of age. *n* = 4 mice per time points. Graphs of EO771 Xenograft tumor volume at 3 weeks after injection in BL/6^C57^ (o) or BL/6^NZB^ (p) females at indicated age. *n* = 5 mice per time points. (q) Heatmap expression analysis of bulk RNAseq from BL/6^NZB^ mice at 3, 11, 14 and 19 months. *n* = 3 mice per time points. (r) Venn diagram of number of genes whose expression follows an oscillation pattern (up/down/up/down) over the aging time course. (s) Top 9 (out of 52) most significant terms from Gene Set Enrichment (GSE) analysis of bimodal genes unique to BL/6^NZB^ mice. Statistics were performed with an ANNOVA followed by a Tukey post test to detect differences between groups. *p < 0.05, **p < 0.005, ***p < 0.0005.

For the analysis in Figure 1r, bulk RNA‐seq was performed on whole mammary glands from BL/6^C57^ and BL/6NZB mice (*n* = 3 biological replicates per time point) at 3, 11, 14, and 19 months of age. Reads were aligned and quantified using BioJupies. Normalized expression values were compiled, and average expression per time point was calculated for each gene. Fold‐changes (relative the global mean) were then computed. Bimodal genes were defined as those exhibiting a non‐linear oscillatory expression pattern characteristic of the BL/6^NZB^ strain: elevated expression at 3 months, decreased at 11 months, increased again at 14 months, and decreased at 19 months (3 > 11 < 14 > 19 m). This filtering identified bimodal genes in BL/6^NZB^ and in BL/6^C57^.

For the analysis in Figure 1s, the 9964 or 601 bimodal genes were fed into “Reactome_2022”, “BioPlanet_2019”, “WikiPathway_2021_Human”, “KEGG_2021_Human”, “Elsevier_Pathway_Collection”, or “MSigDB_Hallmark_2020” using the enrichR package in R. Term hits were kept if they contained the keywords: “cell cycle”, “checkpoint”, “G1/S”, “G2/M”, “retinoblastoma”, “RB”, “p53”, “apoptosis”, “programmed cell death”, “caspase”, “p53”, “DNA repair”, “DNA damage”, “Anti‐proliferative signaling”, or “senescence”. Significance was determined by Fisher's exact test‐based over‐representation analysis (the default in enrichR), not full GSEA with Normalized Enrichment Score (NES). The BL/6^NZB^ Geneset resulted in 49 hits, while the BL/6^C57^ geneset resulted in 0 hits by this analysis.

### 2.8 Single Cell RNA Sequencing and Analysis

Freshly isolated snap frozen Inguinal mammary glands were submitted to GENEWIZ LLC, NJ, USA for nuclei isolation and GEM (Gel Bead‐based Emulsion) single cell RNA seq analysis on the 10× Genomics platform. Cellranger 7.0.1 was used to perform alignment of the raw FASTQ reads, filtering, barcode counting, UMI counting, and generation of the cloupe files used in downstream analysis. The cellranger count command was run with default settings, aligning to the mm10‐2020‐A *Mus musculus* 10× reference genome. The loupe browser was used to identify cells expressing markers published by Brugge and are as follow: Mfge8, Trf, Csn3, Wfdc18, Ltf (Luminal Alveolar (AV) cells), Prlr, Pgr, Esr1, Cited1, Prom1 (Luminal Hormone Sensitive (HS) cells), Krt17, Krt14, and Krt5 (Myoepithelial cells). This browser feature is described here (https://support.10xgenomics.com/single‐cell‐atac/software/visualization/latest/tutorial‐celltypes). No additional code was used. Azenta Life Sciences uses the 10× Genomics Chromium Single Cell Gene Expression kits, which include options for nuclei isolation. Specifically, they have optimized workflows for processing both cells and nuclei with the 10× Genomics Chromium platform. 10× Genomics Chromium Single Cell Gene Expression Flex Kit. This kit is designed for flexibility, allowing the processing of both cells and nuclei. Suitable for a broader range of sample types, including fresh or frozen cells, nuclei from various tissues, and even samples with low viability. The Flex Kit supports the processing of both single‐cell and single‐nuclei RNA sequencing in one workflow but requires additional steps or reagents for nuclei isolation if that's the primary focus. After using the 10× Genomics Chromium Single Cell Gene Expression Flex Kit for nuclei or cell isolation, Azenta Life Sciences follows a detailed library preparation process. After isolating nuclei using the Flex Kit or the Nuclei were reuspended in a suitable buffer. The were combined with barcoded Gel Beads, Master Mix, and Partitioning Oil in the 10× Chromium Controller or Next GEM Chip. This step encapsulates individual nuclei or cells in Gel Beads‐in‐emulsion (GEMs), where each GEM contains one nucleus or cell along with a unique barcode. Reverse transcription occurs inside the GEMs, converting the RNA into barcoded cDNA. cDNA is amplified through PCR. The amplified cDNA was fragmented to an appropriate size for sequencing. The ends of the fragmented cDNA were repaired, and an ‘A’ base is added to facilitate adapter ligation. Unique Dual Index (UDI) Adapters: Adapters with sample‐specific indices were ligated to the cDNA fragments. The library with the sample index adapters was then amplified. A Bioanalyzer was used to check the size distribution and quality of the library, ensuring only high‐quality libraries proceed to sequencing.

### 2.9 Statistics

Statistical analyses were conducted using GraphPad Prism Software. A *p*‐value cutoff of 0.05 was applied to determine significance levels. ns = not significant, * *p* < 0.05, ** *p* < 0.01, *** *p* < 0.001, **** *p* < 0.0001.

Correlations of high and low signature patient groups with clinico‐pathological characteristics were analyzed by Fisher's exact test.

## 3 Results

### 3.1 Multi‐Analyses Show Bimodal Aging of the Mammary Gland in BL/6^NZB^ Females and Windows Favorable to Cancer Formation

After confirming the sequence of their mitochondria genomes (Figure S1), we aged BL/6^C57^ and BL/6^NZB^ female mice at 3, 6, 11, 14 and 19 months (Figure S2a). We selected these time points to include (1) young adult (3, 6 months) at the peak of their reproductive capacity, (2) 11 and 14 months where estrous cycles are irregular and decrease in frequency and (3) 19 months, where estrous cycles are no longer observed (Nelson et al. 1982). Therefore, we reasoned that this age range captures both peaks of incidence of breast cancer in humans (11 m in mice ~45 years in humans and 19 m in mice ~65 years in humans) as well as the decline in ovarian function associated with the menopausal transition with aging in humans. We also elected to analyze: (a) the architecture of the mammary ductal tree, (b) stromal and peri‐ductal collagen and (c) adipocytes, since these parameters have been reported to be altered during aging of the breast in humans (Ghosh, Hartmann, et al. 2010; Ghosh, Vachon, et al. 2010; Milanese et al. 2006; Radisky et al. 2016).

We first compared the mammary glands between genotypes at 3 months. We found that the BL/6^C57^ females have a significantly higher number of mammary branches compared to the BL/6^NZB^ females (Figure S2b,c). The amount of stromal and peri‐ductal collagen was also higher in BL/6^C57^ females (Figure S2d,e). However, these differences were no longer observed between strains at 6 months (Figure S2f–i).

We then compared the mammary glands at different ages within each genotype. We found that the number of mammary branches decreases gradually after the age of 11 months (Figure 1a) in BL/6^C57^ females. However, in BL/6^NZB^ females, an increase was observed at 11 months (Figure 1c), then a decrease was observed between 11 and 14 months followed by an increase at 19 months (Figure 1c), indicating an oscillation pattern. No significant differences in mammary duct thickness were observed between ages in BL/6^C57^ females (Figure 1d) but the same oscillation pattern was observed in BL/6^NZB^ females (Figure 1e). The number of adipocytes was also assessed over aging and we found that the number of adipocytes decreases in BL/6^C57^ females between the age of 11 months and 14 months and remained low at 19 months (Figure 1f,g), while the oscillation pattern was again observed in BL/6^NZB^ females (Figure 1f,h). As an additional morphological feature of the mammary gland, we measured stromal and periductal collagen over aging. No difference was observed in BL/6^C57^ females (Figure 1i–k). However, a highly significant oscillation pattern in stromal collagen staining was observed in BL/6^NZB^ females over aging (Figure 1l,m) and the same trend was also observed for peri‐ductal collagen (Figure 1l,n). Collectively, while the morphological differences in the mammary gland between genotypes over aging were subtle, the reproducibility of the oscillation pattern across analyses supported further investigation into this phenomenon.

Since this oscillation pattern at 11 and 19 months correlates to the peak incidence of breast cancer at 45 and 65 in humans, we next determined if the mammary gland at different ages is more permissive to the growth of exogenous cancer cells using the EO771 murine mammary tumor cell line, which was originally derived from C57BL6 mice. Since this model is an aggressive tumor model, we selected tumor volume at 3 weeks after injection as the endpoint. Using this endpoint, we found that tumors do not form at 3 (Figure 1o,p) or 6 (Figure S3a) months of age in either genotype. Tumors also did not form at 11 months in BL/6^C57^ females but tumor growth was observed at 14 and 19 months (Figure 1o), with significantly larger tumors in 19‐month‐old mice, indicating a progressive increase in tumor formation with age in this model. In the BL/6^NZB^ females however, tumor formation showed an oscillation pattern, with increased tumor formation at 11 and 19 months but no tumor formation at 3 and 14 months (Figure 1p). Since tumor volumes were the highest at 19 months in both genotypes, we repeated the EO771 xenografts in mice aged 22 months or older (22–26 months) in both genotypes and found that tumor volumes were even higher in elderly BL/6^C57^ females but were not observed in BL/6^NZB^ females (Figure S3b). The same observation was made when tumor incidence was recorded (Figure S3c,d). These results therefore indicate that the mammary glands of BL/6^C57^ females are more permissive to tumor formation with increasing age but show a bimodal pattern in the BL/6^NZB^ females. To determine if this differential pattern of aging and tumor formation between genotypes may reflect a difference in estrogen levels, we measured estrogen across ages in both genotypes. We found that in BL/6^C57^ females, a gradual decrease in estrogen is observed between 3, 6 and 11 months but the levels remain constant after 11 months (Figure S4a), a result that is line with a previous report (Nelson et al. 1981). In contrast, the level of estrogen in BL/6^NZB^ females over aging was surprisingly constant (Figure S4a). FSH levels increased over aging in both genotypes (Figure S4b). However, no differences in estrous cycle were observed at 3 and 6 months between strains (Figure S4c,d). The finding of lower estrogen in BL/6^NZB^ females is in fact in agreement with the decreased branching observed at 3 months in BL/6^NZB^ females relative to BL/6^C57^ females, since estrogen is known to be a driver of mammary gland branching at puberty (Bocchinfuso et al. 2000; Daniel et al. 1987; Haslam 1988; Mallepell et al. 2006). Most importantly, since the oscillation pattern is observed starting at 11 months, where estrogen levels are the same between genotypes, this result rules out the possibility that a difference in estrogen levels is the driver of the observed difference in aging patterns of the mammary glands.

Intrigued by the recurrent oscillation pattern of aging in BL/6^NZB^ females, we performed bulk RNAseq on the mammary glands over aging in both genotypes. RNAseq analysis revealed many genes showing an oscillation pattern over aging in the BL/6^NZB^ females, while this was observed to a far lesser extent in BL/6^C57^ females (Figure 1q and Figure S5). To better quantify the number of genes that display an oscillation pattern, we screened the bulk RNAseq dataset for genes that adopt a bimodal pattern (up at 3 months, down at 11, up at 14 months and down at 19 months) over aging and found that 9964 genes (Appendix 1) adopt such pattern and are unique to BL/6^NZB^ females, while only 601 bimodal genes (Appendix 2) were found in BL/6^C57^ females and 636 bimodal genes were shared between genotypes (Figure 1r). Gene Set Enrichment (GSE) analysis for terms related to tumor suppression and cell cycle regulation of bimodal genes unique to BL/6^NZB^ females revealed a total of 49 hits associated with down regulation of tumor suppressor pathways (Figure 1s and Figure S6), while none were observed with the bimodal genes unique to BL/6^C57^ females.

Therefore, the bimodal pattern of aging is observed using bulk RNAseq as well as analyses of the mammary epithelial architecture, adipocytes and collagen staining. To gain more refined and quantitative insights into the oscillation pattern of aging in BL/6^NZB^ females, we next performed both matrix proteomics and single nuclei RNA sequencing of the mammary epithelial cells.

### 3.2 Matrix Proteomic Shows Bimodal Distribution Over Aging of the Mammary Gland in BL/6^NZB^ Females

**FIGURE 2:**
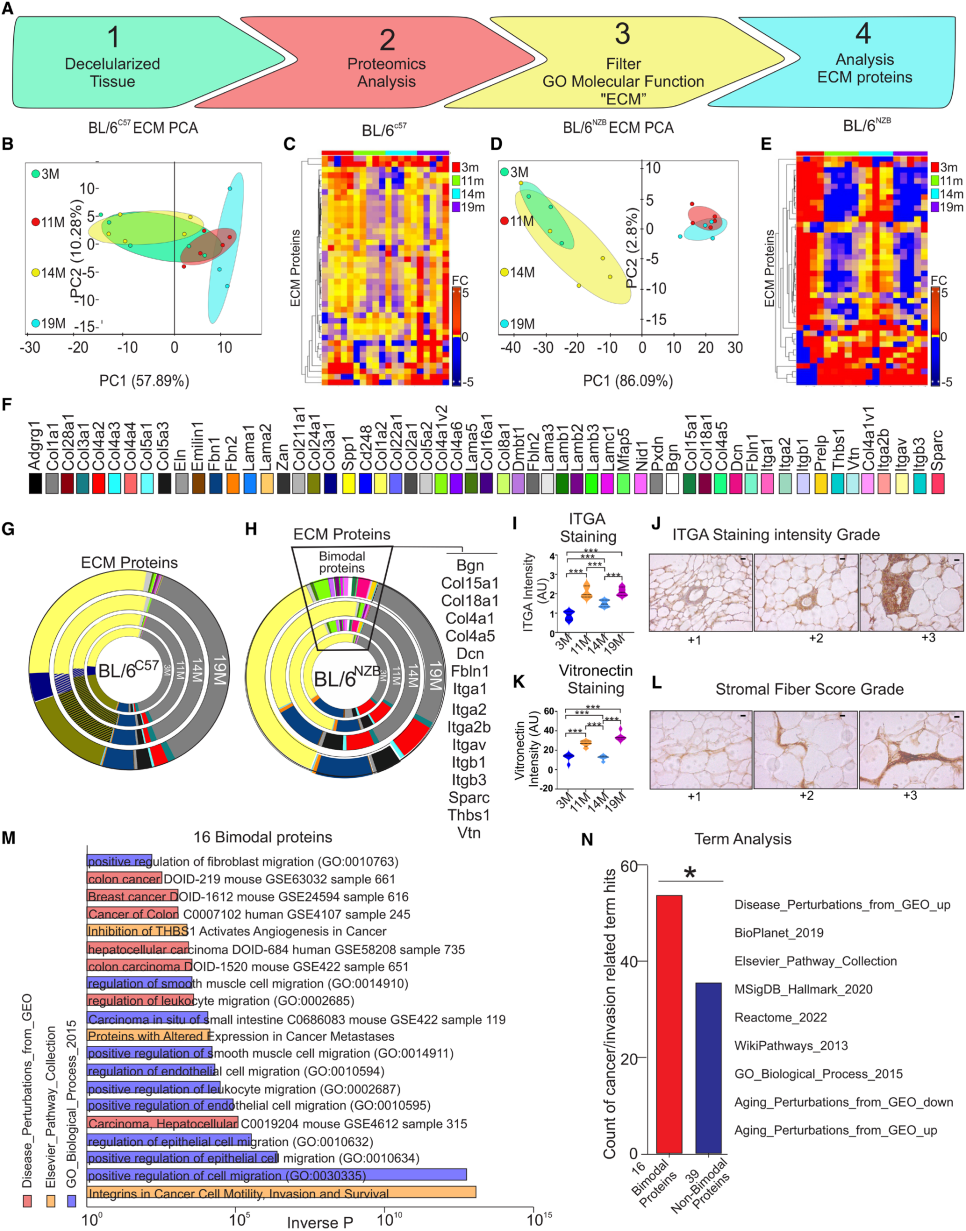
Proteomic analysis of decellularized mammary glands reveals bimodal ECM proteins that are associated with tumor progression in BL/6^NZB^ mice. (A) Schematic representation of sample collection and preparation for subsequent proteomics analysis. Principal component analysis of proteomics analysis from BL/6^C57^ (B) or BL/6^NZB^ (D) mice at 3, 11, 14, or 19 months of age. *n* = 5 mice per time point. Heatmap of proportional representation of ECM proteins from individual BL/6^C57^ (C) and BL/6^NZB^ (E) females at indicated ages. (F) Color code list of ECM proteins used for analysis. Proportional pie graph representation of ECM proteins from BL/6^C57^ (G) or BL/6^NZB^ (H) mice at 3, 11, 14, or 19 months of age. (I) Quantification of ITGA staining intensity in BL/6^NZB^ mice at 3, 11, 14, or 19 months of age. *n* = 5 mice per time point and a minimum of 20 ducts/section were used for scoring. (J) Representative images of ITGA staining and scoring criteria. (K) Quantification of Vitronectin staining intensity in BL/6^NZB^ mice at 3, 11 14, or 19 months of age. *n* = 4 mice per time point and a minimum of 20 ducts were used for scoring (L) Representative images of Vitronectin staining and scoring criteria. (M) Gene Set Enrichment (GSE) analysis of the 16 bimodal ECM proteins indicated in panel E. (N) Term analysis related to cancer or invasion following GSE analysis of either the 16 bimodal or remaining 39 non‐bimodal ECM proteins. *p < 0.05 Fisher's exact *t*‐test.

For proteomics, the mammary glands at all ages and from both genotypes were decellularized and the extracellular matrix (ECM) subjected to unbiased proteomics. The resulting proteins were then filtered to eliminate contaminants by selecting only for proteins known to be associated directly with the ECM, which resulted in the selection of 56 proteins for analysis (Figure 2A). Principal Component Analysis (PCA) of the proteomics from the decellularized mammary glands in the BL/6^C57^ female mice showed no specific clustering between age groups (Figure 2B). Additionally, no oscillation pattern in the composition of ECM proteins was observed (Figure 2C). This result is consistent with a previous report of the analysis of ECM proteins in the mammary gland (Bahcecioglu et al. 2021).

In contrast, in BL/6^NZB^ females, PCA analysis revealed clustering between 3 and 14, as well as 11 and 19 months of age, consistent with the bimodal pattern observed in the other analyses (Figure 2D). Additionally, heat map analysis revealed a striking oscillation pattern in the composition of ECM proteins (Figure 2E). To better visualize the oscillation, we color coded the 56 ECM proteins (Figure 2F) and combined the relative protein abundance of each mouse with respect to age (*n* = 5 per time point) to graph the average change in abundance between age groups. We found a relatively stable distribution of these proteins across ages (Figure 2G and Figure S7) in BL/6^C57^ females. However, in BL/6^NZB^ females, an oscillation in ECM proteins was observed (Figure 2H). Of these oscillating proteins, we selected those that show at least 4‐fold fluctuation between ages. Using this criteria, we identified 16 ECM proteins that are more abundant and common at 11 and 19 months in the mammary glands of BL/6^NZB^ females (Figure 2H and Figure S8). Of these 16 bimodal ECM proteins, we identified 2, ITGA and vitronectin, for which antibodies that recognize the mouse protein are available and are also compatible with analysis by immunohistochemistry. We therefore performed IHC of the two bimodal ECM proteins, which confirmed their bimodal expression over aging (Figure 2I–L).

To further investigate the biological impact of the expression of these proteins, we performed pathway enrichment analysis related to the combined expression of these 16 bimodal proteins as well as the remaining non‐cycling proteins and focused on terms that were uniquely associated with one set or the other. We found that the top 20 pathways uniquely associated with the 16 bimodal proteins related to cancer (Figure 2M). We then compared the number of cancer or invasion related term hits unique to bimodal or non‐bimodal proteins and found that the 16 bimodal proteins correlated with 55 cancer and invasion related hits, while this number was only 38 using the 39 non‐bimodal proteins related to cancer (Figure 2N).

### 3.3 Bimodal Gene Signature Revealed by snRNAseq Analysis Over Aging in BL/6^NZB^ Females and Is Enriched in Breast Cancer Diagnosed at 45 and 65 in Humans

**FIGURE 3:**
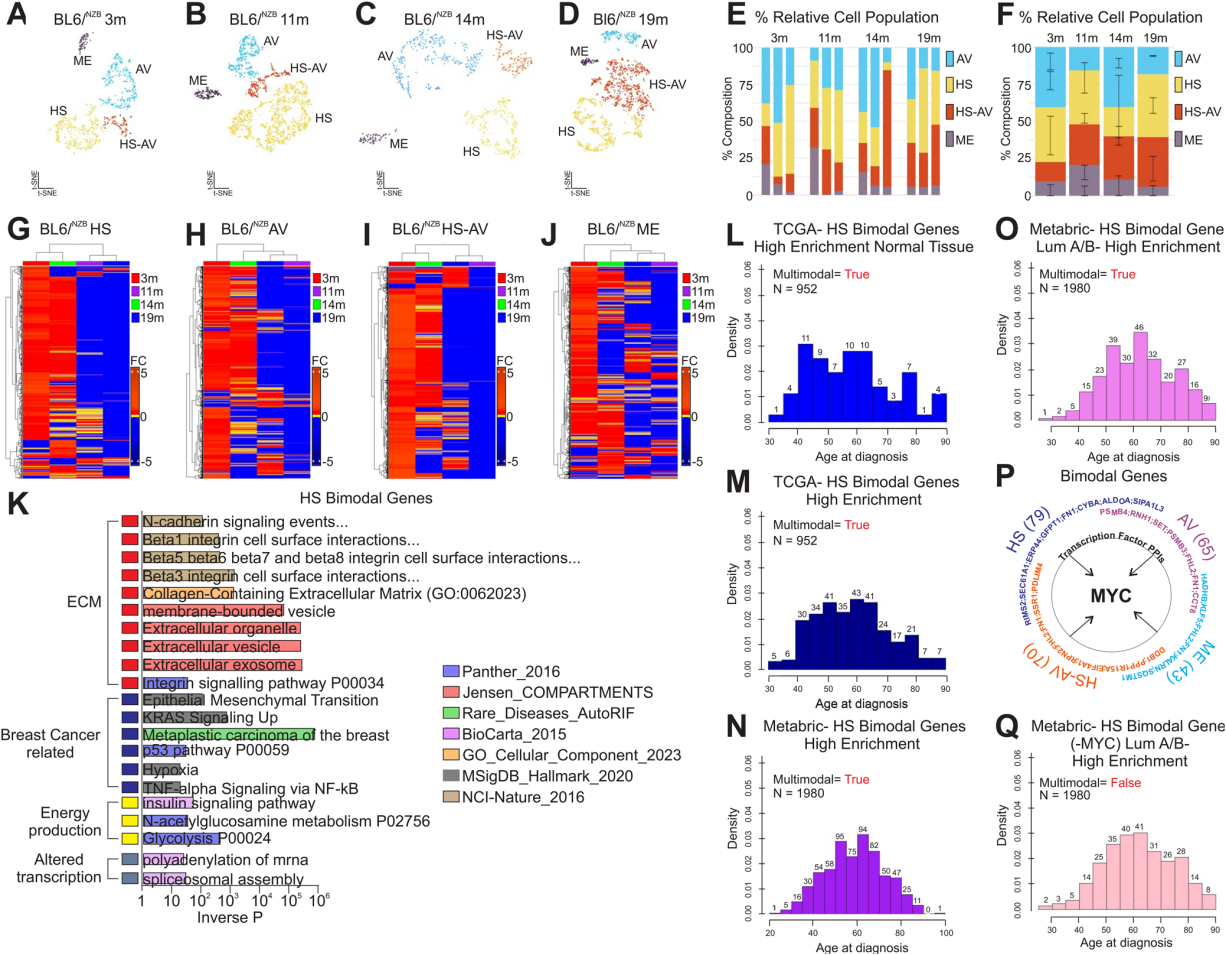
Single nuclei RNAseq analysis shows bimodal fluctuation in the transcriptomes of individual mammary epithelial cell populations in BL/6^NZB^ females over aging. (A) t‐SNE clustergram of single nuclei RNAseq data from 3 BL/6^NZB^ mice at 3 (A), 11 (B) 14 (C), or 19 (D). AV, alveolar cells; HS, Hormone sensitive; HS‐AV, hormone sensitive alveolar cells; ME, myoepithelial cells. (E, F) Proportional representation of HS, AV, HS‐AV, and ME cells in individual mice (E) (*n* = 3 mice per time point, 12 mice total) or combined by time point (F). (G–J) Heatmap and hierarchical clustering of differential gene expression data at indicated ages in HS cells (G), AV cells (H), HS‐AV cells (I), or HS‐AV (J). (K) Gene Set Enrichment analysis of the 79 cycling genes identified in the HS cell population. (L) Histogram of patient age at diagnosis for TCGA patients highly enriched for the bimodal gene signature found in HS cells in normal adjacent tissue. (M) Histogram of patient age at diagnosis for TCGA breast cancer patients highly enriched for the HS bimodal gene signature. (N) Histogram of patient age at diagnosis for all METABRIC breast cancer patients highly enriched for the HS bimodal gene signature. (O) Histogram of patient age at diagnosis for Luminal A/B METABRIC breast cancer patients highly enriched for the HS bimodal gene signature. (P) Protein–Protein Interaction (PPI) analysis of bimodal genes found in HS, AV, HS‐AV, and ME cell populations reveals c‐Myc as a common binding partner. (Q) Histogram of patient age at diagnosis for Luminal A/B METABRIC patients highly enriched for the HS bimodal gene signature minus 13 c‐Myc interacting proteins. Multimodality was assessed using a custom density‐based test, where a kernel density estimate (bandwidth = ‘SJ’) identified significant peaks (local maxima) exceeding a height threshold of 75% of the maximum density, indicating multimodality if multiple peaks were detected. Density values are shown, with counts labeled above each bin.

Having observed bimodal changes in the relative abundance of ECM proteins in the BL/6^NZB^ females, we then performed single nuclei RNAseq analysis on primary epithelial cells derived from BL/6^NZB^ females to gain insight into the changes in cell population over time. Four different types of mammary epithelial cells were identified: the luminal hormonal sensitive cells (HS), the luminal alveolar cells (AV), the mixed hormonal/alveolar population (HS‐AV), which expresses markers of both the HS and the AV cells, and the myoepithelial cells (ME) (Figure 3A–D). Violin plot analysis of markers used to identify specific cell populations confirmed the enrichment in the appropriate cell population (Figure S9). Comparison of the t‐SNE maps between ages indicated a trend in oscillation in cell numbers between ages (Figure 3E,F), however this observation did not reach statistical significance.

We then analyzed the cell‐type specific transcriptome of each cell type according to age. Remarkably, we found that in each population, the gene expression profiles of cells at 11 months and 19 months hierarchically cluster together, while the transcriptomes at 3 months and 14 months hierarchically cluster together (Figure 3G–J and Figure S10). This finding indicates that the transcriptional program of individual cell types in the mammary gland also follows a bimodal oscillation pattern. We then searched for genes that adopt the bimodal pattern of expression (up at both 11 and 19 months and down at 3 and 14 months) over aging in each cell type and found 79 bimodal genes in the luminal HS cells (Figure S11), 65 bimodal genes in the AV cells (Figure S12), 43 bimodal genes in the ME cells (Figure S13) and 70 bimodal genes in the mixed lineage of HS‐AV cells (Figure S14). Because most breast cancers are estrogen receptor alpha (ERα) positive and the HS cells are characterized by ERα positivity, we chose to focus on the HS signature for further validation. To interrogate the role of the bimodal genes, we performed pathway analysis of the 79 bimodal genes specifically expressed in the luminal HS cells across multiple databases. Focusing on the most significant hits, we found that these bimodal genes are enriched in breast cancer‐related pathways (6) and ECM interaction related pathways (10) (Figure 3K and Figure S15a), while non‐bimodal genes in the HS cells showed no ECM related pathways and only one breast cancer related pathway (Figure S15b). Further, analysis of the HS bimodal gene signature in the bulk RNAseq data revealed an oscillation of expression in the BL/6^NZB^ (Figure S16) but not in the BL/6^C57^ females (Figure S17). Gene set analyses of the other cell specific bimodal signatures also revealed cancer related pathways (Figures S18–S20). Combined with the data from the ECM proteomics, these results support the notion that bimodal aging is associated with pro‐tumorigenic windows at 11 and 19 months.

Since the incidence of breast cancer shows a bimodal age distribution at 45 and 65 (Allott et al. 2020; Anderson et al. 2006; Desai and Guddati 2022) (the equivalent of 11 and 19 months in mice), our data raise the possibility that if applicable to humans, bimodal aging would increase the predisposition to develop breast cancer during these age windows. If so, we reasoned that the bimodal gene signature should be enriched in breast cancer patients diagnosed around the age of 45 and 65 years. Importantly, this enrichment should not be tumor‐specific but also found in the normal adjacent breast tissue since the signature in mice was identified in normal mammary gland. We therefore analyzed data from 952 primary breast cancers in the TCGA breast cancer dataset and identified 108 patients for which data are available for both the tumor and normal adjacent tissue from the same patient. We performed single‐sample gene set enrichment analysis (ssGSEA) to generate an enrichment score for each patient in the TCGA‐BRCA cohort (primary tumor samples) for the HS bimodal gene signature identified in mice. We divided the cohort into High enrichment score (top tertile) and Low enrichment score (bottom tertile) groups based on these scores.

Analysis of patient age at diagnosis among normal adjacent tissue samples revealed a multimodal distribution (Figure 3L) among highly enriched samples. We found a similar multimodal distribution of patient age at diagnosis across the highly enriched patients with peaks at 45 and 65 (Figure 3M), while the age distribution of patients classified within Low enrichment score showed a unimodal distribution with a peak at 65 (Figure S21). Patients with High enrichment score were more likely to be younger but not associated with BRCA1 mutation (< or equal to 43 y.o.) (Figure S22).

To further validate this result, we repeated the analysis in the METABRIC breast cancer dataset and found that in this larger dataset, the same multimodal distribution is observed (Figure 3N). Since this bimodal gene signature was detected in the hormone sensitive epithelial population, we also performed the analysis focusing on the luminal A and B breast cancer sub‐type and confirmed the bimodal distribution (Figure 3O).

Finally, to gain insight into what may be driving these oscillations, we took advantage of the fact that bimodal signatures are observed in all epithelial cell types. We performed enrichment analysis using the Transcription_Factor_PPIs library in Enrichr to identify transcription factors whose protein–protein interaction partners overlap with bimodal gene sets that we identified in all four mammary epithelial populations. We found that c‐Myc consistently ranked among the top ten hits across all four populations, suggesting it may form regulatory complexes potentially associated with the bimodal expression patterns we observed, and therefore may contribute to the breast cancer risk windows observed in the TCGA‐BRCA single‐sample GSEA (ssGSEA) analysis, where high enrichment scores correlated with multimodal patient age‐at‐diagnosis distributions (Figure 3P).

To determine if c‐Myc impacts the bimodal oscillation, we repeated the ssGSEA of the bimodal HS gene set but subtracted 13 genes known to be driven by or interact with c‐Myc (Figure S11, genes indicated in red). We found that removal of the 13 c‐Myc‐related genes from the HS bimodal gene signature abolished the multimodal distribution (Figure 3Q) among highly enriched patients. This observation suggests that c‐Myc is likely to play a role in the bimodal oscillation observed, at least in the HS cells.

## 4 Discussion

Non‐linear aging, identified using human plasma proteomics, is supported by the observation of waves of changes around the ages of 44 and 60 (Fehlmann et al. 2020; Lehallier et al. 2019; Shen et al. 2024). However, how these waves are associated with specific diseases remains unclear. Since breast cancer shows a bimodal incidence at 45 and 65 (Allott et al. 2020; Anderson et al. 2006; Desai and Guddati 2022), breast cancer stands out as a disease that may be directly affected by non‐linear aging. The current study supports this possibility.

Our results suggest that the bimodal aging pattern, observed in BL/6^NZB^ female mice, creates aging windows that share several features both in terms of composition of the extracellular matrix and epithelial transcriptomes. These features are associated with cancer‐related pathways and the establishment of a host environment that is more permissive to the growth of exogenous cancer cells. By extrapolation to human, the phenomenon described here may contribute to the peak in incidence observed at 45 and 65. However, since datasets with matched human proteomics for normal breast/mammary tissue across relevant timepoints are not currently available, the validation of the bimodal ECM proteins identified here in mice is a limitation and future human proteomics efforts (e.g., through the Human Proteome Map or targeted MS studies) will be required to fully translate the ECM findings.

However, since two‐thirds of breast cancer occur in women over the age of 50 (Benz 2008) and we show that both BL/6^C57^ and BL/6^NZB^ females have the highest level of tumor formation at 19 months, we propose that the increased incidence of tumor formation in the later peak observed at 65 may be due to the additive effect of bimodal and progressive aging patterns.

Equally important is the finding that bimodal aging involves the phase at 14 months, in between the two cancer‐susceptibility phases at 11 months and 19 months. What events and signaling pathways initiate this transition from the 11 months to the 14 months and its reversal between 14 and 19 months represent a key unanswered question. The identification of c‐Myc as a potential driver of the bimodal signature in our study offers an initial clue into the direction for future research aimed at understanding what is likely to be a complex and multi‐layered mechanism involving both the stroma and the epithelial cells. Further, since ROS regulates c‐Myc (Dang et al. 2005; Purhonen et al. 2023) and differences in ROS over aging were observed between BL/6^C57^ and BL/6^NZB^ mice (Latorre‐Pellicer et al. 2016), fluctuation in ROS may impact c‐Myc expression and contribute to the aging pattern observed here. More specifically, ROS levels were found to increase with aging in BL/6^C57^ model, while in the BL/6^NZB^ model, ROS levels were slightly higher in young mice but did not increase with age. Therefore, it is possible that fluctuation in ROS levels at 3, 11, 14 and 19 months may contribute to the pattern of aging observed here.

Another important question that the discovery of bimodal aging raises is whether it also takes place in other organs in BL/6^NZB^ mice and whether it affects males as well.

BL/6^NZB^ mice show increased longevity (Latorre‐Pellicer et al. 2016). However, differences in the speed of aging are observed between organs and only a subset of aging organs is associated with longevity (Oh et al. 2025). Therefore, how organismal longevity impacts the aging of the mammary gland observed in the current study is unknown. We will actively seek to investigate these possibilities in the future.

Our study is timely since the concept of non‐linear changes during aging, using samples derived from human blood, has been reported by a number of independent studies (Fehlmann et al. 2020; Lehallier et al. 2019; Shen et al. 2024) and consistently describes waves or crests of changes around the ages of 44 and 60. However, in one study, the proteins found in these waves are reported to be largely non‐overlapping (Lehallier et al. 2019), but in another study, significant overlap between waves was observed (Shen et al. 2024). While technical differences in proteomics acquisition data platforms between studies may provide an explanation for this discrepancy, it remains formally possible that these results reflect fundamental differences in the aging patterns that are being captured by the different cohorts used between studies rather than merely technical differences. This possibility is supported by the observation that wide variability is observed between chronological aging and biological aging among individuals.

In summary, our findings support the idea that two distinct patterns of aging of the mammary gland exist, therefore opening the possibility that specific aging patterns may contribute to the observed bimodal age distribution of incidence of breast cancer. Further, our study adds to the growing evidence of non‐linear aging.

## Author Contributions

E.C.J. analyzed the proteomics, bulk and single cell RNAseq data and has generated data in Figures 1q–s and 2C,E,G,H,M,N and all of Figure 3. He also generated all Figures S4–S19. M.C. has generated data included in Figure 1a–p; Figure 2I–L; Figures S1–S3 and helped colony management and mammary gland analysis. T.M. has generated data included in Figure 1a–p and Figure S1a–c. M.T.‐M. is a bioinformatician that contributed to the analysis in Figure 3. D.S. has generated the enrichment score and perform some of the analysis in human data in Figure 3 as well as Figure S20. I.B. has provided access the METABRIC dataset. D.G. has guided the project, interpreted the data and wrote the manuscript.

## Funding

This work is supported by an NIH R01AG075589 to D.G. and a grant from the Samuel Waxman Cancer Research Foundation.

## Conflicts of Interest

The authors declare no conflicts of interest.

## Supporting information


**Figure S1:** Sequence and comparison of the mitochondria genomes in BL/6C57 and BL/6NZB mice. Variants are organized by mitochondrial respiratory complex and genomic subunit and were identified by whole mitochondrial genome next‐generation sequencing (NGS) performed on animals used in this study. All variants were confirmed to match the expected strain‐specific haplotype reported by Latorre‐Pellicer et al. (Latorre‐Pellicer et al. 2016). Nucleotide positions refer to the C57BL/6 mitochondrial reference genome.
**Figure S2:** Experimental design and analysis of mammary gland at base line at 3 and 6 months. (a) Schematic representation of the BL/6C57 and BL6/NZB model and experimental design. (b) Representative images of mammary gland whole mount from BL6/C57 or BL6/NZB at 3 months of age. (c–e) Analysis of mammary gland whole mounts in indicated genotype at 3 months for number of branches (c), stromal collagen (d) and periductal collagen (e). (f) Representative images of mammary gland whole mount from BL6/C57 or BL6/NZB at 6 months of age. (g–i) Analysis of mammary gland whole mounts in indicated genotype at 6 months for number of branches (g), stromal collagen (h) and periductal collagen (i). Statistical differences were assessed with Student's *t*‐test.
**Figure S3:** Tumor formation at 6 and 24 months. Graphs of tumor xenograft volume from mice at either 6 (a) or 24 (b) months of age. Graph a tumor incidence in BL/6C57 and BL/6NZB mice. Tumor incidence (%) indicates the percentage of mice in each group that developed tumors relative to the total number of mice in that group. Statistical differences were assessed with Student's *t*‐test.
**Figure S4:** Estrogen levels an estrous cycle. Graph of serum estradiol (a) and FSH (b) levels measure by ELISA in BL6/C57 or BL6/NZB females at the ages indicated. Differences were assessed by ANNOVA followed by a Tukey post test. ****p < 0.00005. (c) Vaginal smears from BL6/C57 and BL6/NZB females at 3 months of age. cd Vaginal smears from BL6/C57 and BL6/NZB females at 6 months of age.
**Figure S5:** Heatmap of bulk RNAseq from BL6/C57 mice at 3, 11, 14, or 19 months of age. *n* = 3 mice per time point.
**Figure S6:** List of 49 hits term shown in Figure 1s. Raw *p* value and Adjusted *p* value (Benjamini‐Hochberg FDR correction, standard in EnrichR) along with Overlap: Number of input genes in the term/total genes in the term along with Odds. Ratio and Combined. Score (log odds * −log10(P)) for ranking strength are shown.
**Figure S7:** Average proportional values of ECM proteins measured by proteomics analysis from BL6/C57 mice at 3, 11, 14, or 19 months of age. *n* = 5 mice per time point.
**Figure S8:** Average proportional values of ECM proteins measured by proteomics analysis from BL6/NZB mice at 3, 11, 14, or 19 months of age. *n* = 5 mice per time point.
**Figure S9:** Violin plots of the Log2 Sum Expression values of the markers genes for myoepithelial (ME) (a), alveolar cells (AV) (b), hormone sensitive cells (HS) (c), or hormone sensitive alveolar cells (HS‐AV) (d).
**Figure S10:** Gene expression heatmaps and hierarchical clustering of snRNAseq analysis of hormone sensitive (HS) cells (a), alveolar cells (AV) (b), hormone sensitive alveolar cells (HS‐AV) (c), or Myoepithelial cells (ME) (d) from individual females (*n* = 3 per age).
**Figure S11:** Bimodal (down at 3 m, up at 11 m, down at 14 m, up at 19 m) genes found in the hormone sensitive cell (HS) population. Red text indicates genes associated with c‐MYC.
**Figure S12:** Bimodal (down at 3 m, up at 11 m, down at 14 m, up at 19 m) genes found in the alveolar cell (AV) population. Red text indicates genes associated with c‐MYC.
**Figure S13:** Bimodal (down at 3 m, up at 11 m, down at 14 m, up at 19 m) genes found in the Myoepithelial cell (ME) population. Red text indicates genes associated with c MYC.
**Figure S14:** Bimodal (down at 3 m, up at 11 m, down at 14 m, up at 19 m) genes found in the hormone sensitive alveolar cell (HS‐AV) population. Red text indicates genes associated with c‐MYC.
**Figure S16:** Heatmap of the HS bimodal genes signature in the bulk RNAseq data from BL/6NZB mice at 3, 11, 14, or 19 months of age. *N* = 3 mice per time point.
**Figure S17:** Heatmap of the HS bimodal genes signature in the bulk RNAseq data from BL/6C57 mice at 3, 11, 14, or 19 months of age. *N* = 3 mice per time point.
**Figure S18:** Gene set enrichment analysis of the bimodal genes found in the alveolar cell (AV) population.
**Figure S19:** Gene set enrichment analysis of the bimodal genes found in the hormone sensitive alveolar cell (HS‐AV) population.
**Figure S20:** Gene set enrichment analysis of the bimodal genes found in the myoepithelial cell (ME) population.

## Data Availability

The data that support the findings of this study are openly available in Gene expression omnibus at https://www.ncbi.nlm.nih.gov/geo/query/acc.cgi?acc=GSE275925, reference number GSE275925.
