# Supplementary material for: Mitochondria Dictate Progressive Versus Bimodal Aging of the Mammary Gland
Source: Aging Cell. 2026 Aug 9;25(8):e70661. doi: 10.1111/acel.70661 (PMC13454215; doi:10.1111/acel.70661)
Supplement: Supplementary file 1 [file ACEL-25-e70661-s001.pdf]

SUPPLEMENTARY FIGURES

Mitochondrial DNA sequence variants distinguishing NZB and C57BL/6 mouse strains

Gene / Subunit	mtDNA Position	C57BL/6	NZB	Variant Class
**Non-coding Regions**				
tRNA-Phe	55	G	A	Non-coding
rRNA 16S	716	A	G	Non-coding
rRNA 16S	1353	A	G	Non-coding
rRNA 16S	1519	G	A	Non-coding
rRNA 16S	1590	G	A	Non-coding
rRNA 16S	1822	T	C	Non-coding
rRNA 16S	2201	T	C	Non-coding
rRNA 16S	2340	G	A	Non-coding
rRNA 16S	2525	C	T	Non-coding
tRNA-Cys	5204	A	AG	Non-coding
tRNA-Gly	9391	A	G	Non-coding
**NADH Dehydrogenase — Complex I**				
ND1	2766	A	G	Missense
ND1	2767	T	C	Missense
ND1	2798	C	T	Silent
ND1	2814	T	C	Silent
ND1	2840	C	T	Silent
ND1	2934	C	T	Missense
ND1	3194	T	C	Silent
ND1	3260	A	G	Silent
ND1	3422	T	C	Silent
ND1	3467	T	C	Silent
ND1	3599	T	C	Silent
ND1	3692	A	G	Silent
ND2	3932	G	A	Missense
ND2	4123	C	T	Silent
ND2	4276	G	A	Silent
ND2	4324	T	C	Silent
ND2	4408	G	A	Silent

ND2	4706	A	G	Missense
ND2	4732	C	T	Silent
ND2	4771	T	C	Silent
ND2	4885	A	C	Silent
ND2	4903	T	G	Silent
ND3	9461	T	C	Silent
ND3	9530	C	T	Silent
ND3	9581	C	T	Silent
ND3	9599	A	G	Silent
ND4l	9985	G	A	Missense
ND4	10547	C	T	Silent
ND4	10583	A	G	Silent
ND4	10952	C	A	Missense
ND5	11843	G	A	Silent
ND5	11846	C	T	Silent
ND5	11933	A	C	Silent
ND5	12353	C	T	Silent
ND5	12575	T	A	Silent
ND5	12695	A	G	Silent
ND5	12835	T	C	Missense
ND5	12890	A	G	Silent
ND5	13004	G	A	Silent
ND5	13444	C	T	Missense
ND6	13612	T	C	Silent
ND6	13689	C	T	Missense
ND6	13780	T	C	Missense
ND6	13781	A	G	Missense
ND6	13837	A	G	Silent
ND6	13983	A	G	Silent
**Cytochrome c Oxidase — Complex IV**				
COI	5463	G	A	Missense
COI	5552	T	C	Silent
COI	5930	G	A	Silent
COI	6041	T	C	Silent

COI	6407	C	T	Silent
COI	6470	A	G	Silent
COI	6575	C	T	Silent
COI	6620	G	A	Silent
COI	6785	G	A	Silent
COII	7411	A	G	Silent
COIII	8858	T	C	Silent
COIII	8864	C	T	Silent
COIII	9137	A	G	Silent
COIII	9152	T	C	Silent
**ATP Synthase — Complex V**				
ATP8	7870	G	A	Silent
ATP6	8439	A	G	Silent
ATP6	8467	T	C	Silent
ATP6	8568	C	T	Silent
**Cytochrome bc$_1$ Complex — Complex III**				
CYTB	14186	T	C	Silent
CYTB	14211	G	A	Missense
CYTB	14363	A	G	Silent
CYTB	14642	G	A	Silent
CYTB	14738	C	T	Silent
**Control Region**				
D-Loop	15499	T	A	Intergenic
D-Loop	15549	C	T	Intergenic
D-Loop	15578	A	T	Intergenic
D-Loop	15588	C	T	Intergenic
D-Loop	15603	C	T	Intergenic
D-Loop	15657	T	C	Intergenic
D-Loop	15916	C	T	Intergenic
D-Loop	16017	A	C	Intergenic
D-Loop	16268	A	G	Intergenic
D-Loop	16272	T	C	Intergenic

Suppl. Fig. 1. Sequence and comparison of the mitochondria genomes in BL/6^{C57} and BL/6^{NZB} mice. Variants are organized by mitochondrial respiratory complex and genomic subunit and were identified by whole mitochondrial genome next-generation sequencing (NGS) performed on animals used in this study. All variants were confirmed to match the expected strain-specific haplotype reported by Latorre-Pellicer et al. (*Nature*, 2016). Nucleotide positions refer to the C57BL/6 mitochondrial reference genome.

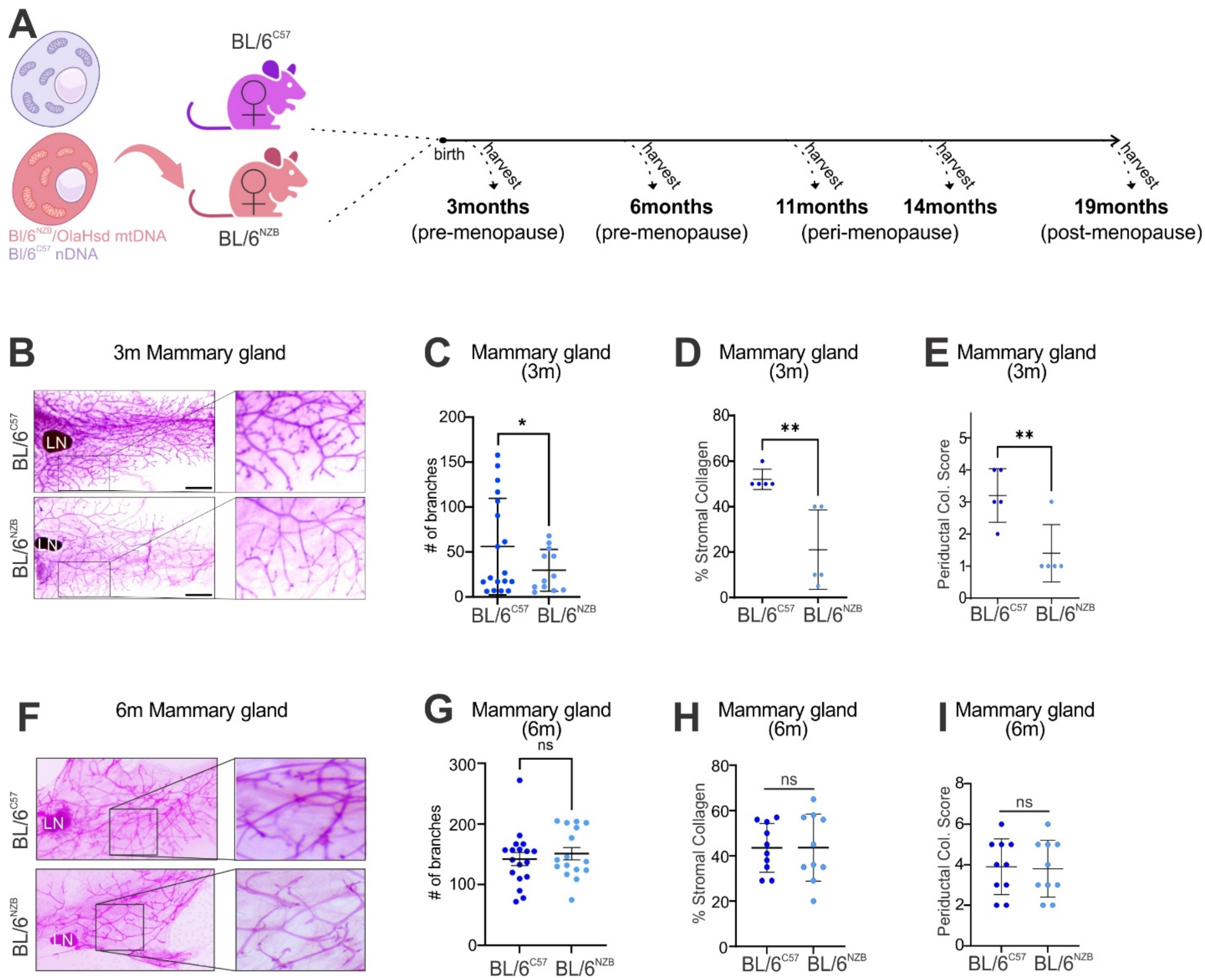

Suppl. Fig. 2. Experimental design and analysis of mammary gland at base line at 3 and 6 months. a) Schematic representation of the BL/6^{C57} and BL6/^{NZB} model and experimental design. b) Representative images of mammary gland whole mount from BL6/^{C57} or BL6/^{NZB} at 3 months of age. c-e) Analysis of mammary gland whole mounts in indicated genotype at 3 months for number of branches (c), stromal collagen (d) and periductal collagen (e). f) Representative images of mammary gland whole mount from BL6/^{C57} or BL6/^{NZB} at 6 months of age. g-i) Analysis of mammary gland whole mounts in

indicated genotype at 6 months for number of branches (g), stromal collagen (h) and periductal collagen (i). Statistical differences were assessed with Student's t-test.

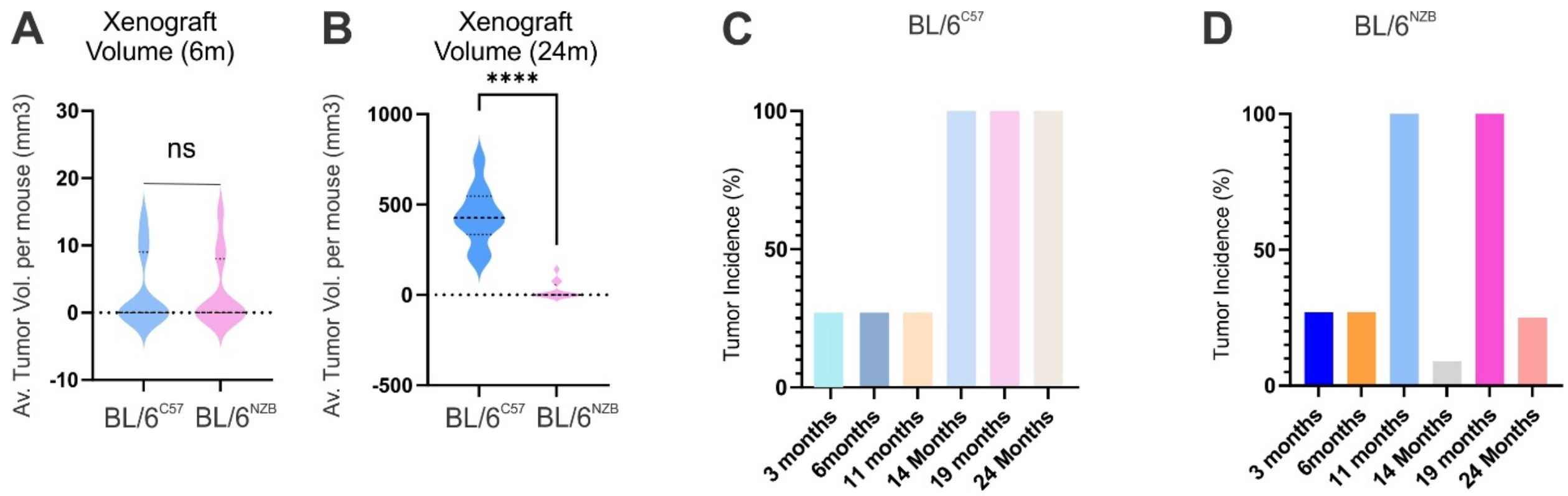

Suppl. Fig. 3. Tumor formation at 6 and 24 months. Graphs of tumor xenograft volume from mice at either 6 (a) or 24 (b) months of age. Graph a tumor incidence in BL/6^{C57} and BL/6^{NZB} mice. Tumor incidence (%) indicates the percentage of mice in each group that developed tumors relative to the total number of mice in that group. Statistical differences were assessed with Student's t-test.

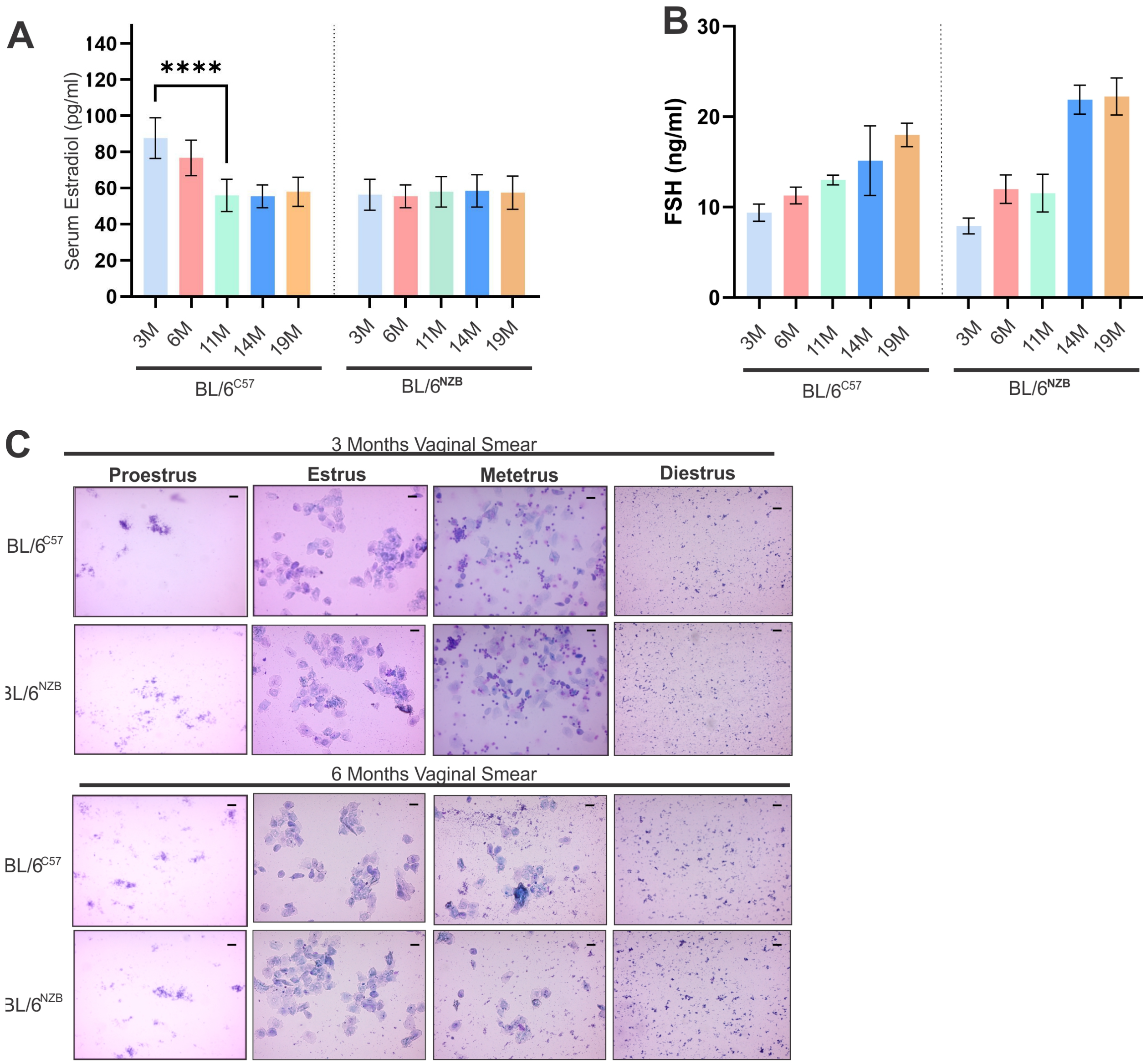

Suppl. Fig. 4. Estrogen levels an estrous cycle. Graph of serum estradiol (a) and FSH (b) levels measure by ELISA in BL6/^{C57} or BL6/^{NZB} females at the ages indicated. Differences were assessed by ANNOVA followed by a Tukey post test. ****p<0.00005. c) Vaginal smears from BL6/^{C57} and BL6/^{NZB} females at 3 months of age. cd Vaginal smears from BL6/^{C57} and BL6/^{NZB} females at 6 months of age.

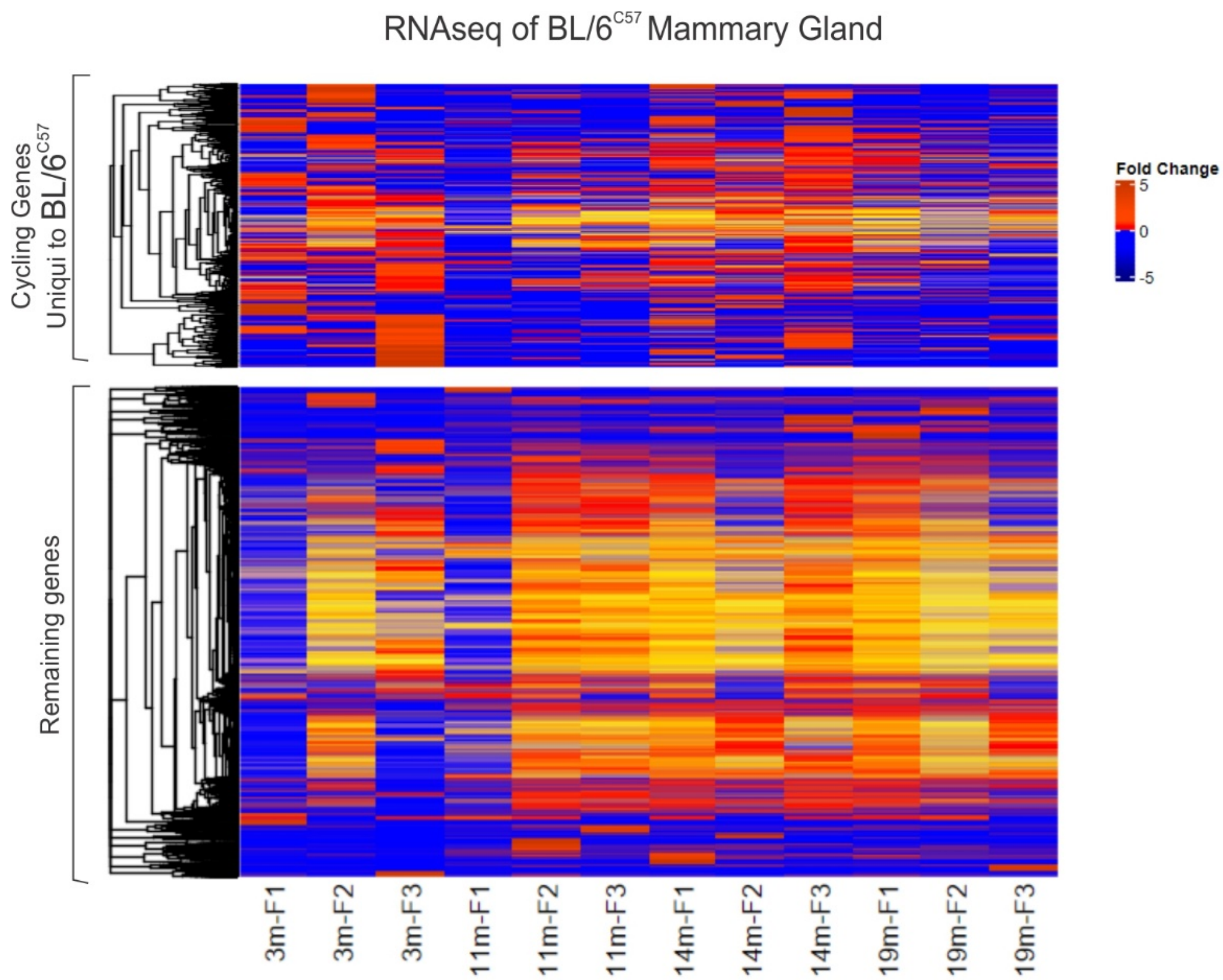
RNAseq of BL/6^C57 Mammary Gland
Cycling Genes Uniqui to BL/6^C57
Remaining genes
Fold Change
5
0
-5
3m-F1
3m-F2
3m-F3
11m-F1
11m-F2
11m-F3
14m-F1
14m-F2
14m-F3
19m-F1
19m-F2
19m-F3

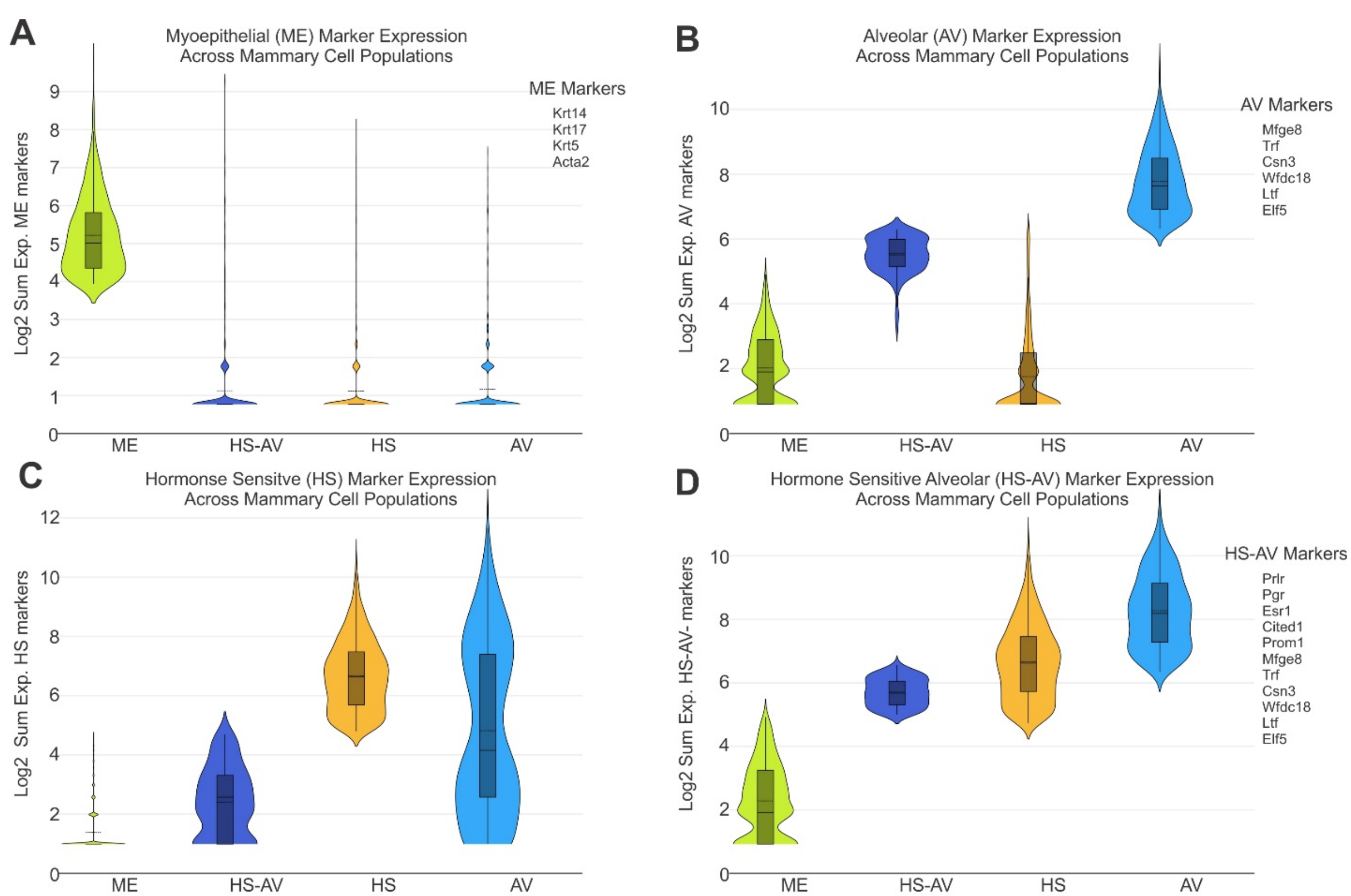
A
Myoepithelial (ME) Marker Expression
Across Mammary Cell Populations
ME Markers
Krt14
Krt17
Krt5
Acta2
Log2 Sum Exp. ME markers
ME
HS-AV
HS
AV
B
Alveolar (AV) Marker Expression
Across Mammary Cell Populations
AV Markers
Mfge8
Trf
Csn3
Wfdc18
Ltf
Elf5
Log2 Sum Exp. AV markers
C
Hormonse Sensitve (HS) Marker Expression
Across Mammary Cell Populations
Log2 Sum Exp. HS markers
D
Hormone Sensitive Alveolar (HS-AV) Marker Expression
Across Mammary Cell Populations
HS-AV Markers
Prlr
Pgr
Esr1
Cited1
Prom1
Mfge8
Trf
Csn3
Wfdc18
Ltf
Elf5
Log2 Sum Exp. HS-AV- markers

Suppl. Fig. 5. Heatmap of bulk RNAseq from BL6/^{C57} mice at 3,11, 14, or 19 months of age. n=3 mice per time point.

Term	Overlap	P.value	Adjusted.P.value	Odds.Ratio	Combined.Score	Genes	Database
G2-M Checkpoint	134/200	3.74E-05	0.000934922	1.810705951	18.45819741	CCNT1;CCNF;HNRNPU;SMC4;SMC2;CCND1;SNRPD1;MYC;KIF5B;CDC27;NUSAP1;NEK2;KMT5A;HMGN2;TMPO;TLE3;SMARCC1;HUS1;LIG3;PRPF4B;CDC25A;CDC25B;TRAIP;ILF3;EWSR1;DKC1;NCL;SRSF2;NUP98;KI	MSigDB_Hallmark_2020
SUMOylation Of DNA Damage Response And Repair Proteins R-HSA-3108214	56/76	0.000172196	0.010308781	2.490895296	21.5882883	MDC1;NUP107;NUP188;SMC5;SMC6;SMC3;HERC2;SUMO1;NUP62;SCMH1;SUMO2;PMEPA1;NDC1;NUP214;PIAS4;SEC13;NUP133;NUP210;PARP1;RING1;NSMCE2;SMC1A;PIAS2;PIAS1;RNF168;NSMCE4A;NUP50;NUI	Reactome_2022
Mitotic Spindle Checkpoint R-HSA-69618	76/110	0.000418538	0.018828293	1.989335301	15.47452753	ERCC6L;AHCTF1;NUP107;ZWILCH;BUB1B;PPP1CC;CDC23;XPO1;PPP2R1A;PPP2R5E;CDC26;CDC27;SPDL1;NDEL1;SEC13;ANAPC7;NUP133;UBE2E1;PPP2R5B;PPP2R5A;PPP2R5C;RANGAP1;CKAP5;DYNC1LI1;DYNC	Reactome_2022
DNA Repair and Chromatin Remodeling Block in Pancreatic Neoplasms	27/33	0.000551196	0.066268751	3.99820297	30.0001937	SMARCD1;SMARCD2;SMARCB1;BRCA2;MYSM1;BRIP1;ARID2;SMARCC1;SMARCC2;ACTL6B;PARP1;PARP2;ACTL6A;ATRX;ARID1A;MLH1;PALB2;SMARCA4;MSH6;KAT2B;KAT2A;RAD50;RAD51;TADA2A;MSH2;MSH3;TA	Elsevier_Pathway_Collection
ERBB1 downstream pathway	73/106	0.000625794	0.026533671	1.968361299	14.51959567	ATF1;USP6NL;ATF2;NCKAP1;PPP2R2A;PIK3CB;WASL;ELK1;EPS8;RPS6KA4;RPS6KA3;PPP2R1A;CAPN2;MLST8;AKT1;RAC1;YWHAH;ACTR3;MAP2K4;ACTR2;MAP2K1;MAP2K2;PDPK1;PRKCD;GAB1;PRKCA;YWHAZ;DU	BioPlanet_2019
Cell cycle	274/453	0.000699179	0.028821709	1.366921055	9.931506913	NUP107;CCNH;CCP110;PPP2R2A;SMC3;PSMD9;PSMD6;CDC23;PSMD5;PPP2R1A;MYC;CDC26;CHEK2;PSMD3;CDC27;BTRC;SKP2;CDK5RAP2;TINF2;HUS1;CSNK1D;CSNK1E;PSME3;ZW10;PSME4;AKAP9;PSME1;PSI	BioPlanet_2019
Cell cycle	83/124	0.001103306	0.05847521	1.801489806	12.26714447	GSK3B;CDKN1B;MCM7;CCNH;BUB1B;SMC3;CDC14A;CDC14B;CCND3;CCND2;CDC23;CCND1;CDC26;CHEK2;MYC;CDC27;EP300;SKP2;YWHAH;ANAPC7;SMC1A;YWHAZ;CDC25A;CDC25B;RBX1;CCNA2;RBL2;DBF4;I	KEGG_2021_Human
Cell Cycle R-HSA-1640170	385/654	0.001236588	0.037015216	1.279347195	8.565739808	MDC1;ANKLE2;NUP107;SMC3;SMC4;PSMD9;PSMD6;PSMD5;MYC;AKT3;PSMD3;AKT1;CDK5RAP2;NUP214;NUP210;PRKCB;CSNK2A2;HUS1;PRKCA;EML4;DYNC1LI1;DYNC1LI2;PSME3;PSME4;PSME1;PSME2;NUP98;	Reactome_2022
Apoptosis Evasion in Cancer: Overview	72/106	0.00124446	0.092522869	1.883917397	12.60162494	HRK;APP;FAF1;TRADD;AATF;BBC3;CASP9;IKBKB;CASP8;AIFM1;CASP6;CASP4;TNFSF10;AKT1;CASP2;MAP3K7;SPTAN1;BOK;MAP2K4;DFFB;ENDOG;APAF1;PARP1;DAPK1;MITF;TRAF2;NGF;YWHAZ;TNFRSF1A;ARHGE	Elsevier_Pathway_Collection
Apoptosis	54/77	0.001611998	0.110260678	2.087574931	13.42369288	APP;FAF1;TRADD;BBC3;CASP9;CASP8;AIFM1;CHEK2;CASP4;TNFSF10;AKT1;CASP2;MAP3K7;SPTAN1;BOK;EDARADD;DFFB;ENDOG;APAF1;PARP1;DAPK1;TRAF2;NGF;TNFRSF1A;GAS2;BIRC5;BIRC2;BIRC3;ROCK1;DI	Elsevier_Pathway_Collection
Mitotic G2-G2/M phases	60/87	0.001749879	0.055251501	1.976175416	12.54517378	CEP57;NUMA1;CCNH;CEP164;CCP110;ACTR1A;PCM1;XPO1;PPP2R1A;CNTRL;CEP250;NEK2;CEP290;NINL;CDK5RAP2;OFD1;HSP90AA1;CSNK1D;CSNK1E;TUBG1;CKAP5;CDC25A;HAUS2;CDC25B;CCNA2;AKAP9;CEI	BioPlanet_2019
ERBB signaling pathway	64/94	0.002070821	0.062716298	1.897241205	11.72459028	GSK3B;CDKN1B;ARAF;CBLC;PIK3CB;ELK1;PIK3CG;CRKL;CCND1;MYC;AKT3;NCK2;AKT1;NCK1;MAP2K4;HSP90AA1;MAP2K1;MAP2K2;PRKCB;GAB1;NRG1;PRKCA;NRG2;BTC;PIK3CA;RAF1;SOS1;CRK;SOS2;CAMK2D	BioPlanet_2019
Frizzled Receptors -> ARRB1/ARRB2 non-Canonical Signaling	15/17	0.00251885	0.138943018	6.658951346	39.84685069	MAP2K4;MAP3K1;ROCK1;ROCK2;LRP5;ARRB1;ARRB2;RHOA;LRP6;MAPK8;DAAM1;DVL1;RAC1;MAP2K7;MAP3K11	Elsevier_Pathway_Collection
Cell cycle WP179	79/120	0.002905921	0.088921175	1.714019116	10.01159421	GSK3B;CDKN1B;MCM7;CCNH;SMC3;CDC14A;CDC14B;CCND3;CCND2;CDC23;CCND1;MYC;CHEK2;CDC27;EP300;SKP2;YWHAH;ANAPC7;SMC1A;YWHAZ;CDC25A;CDC25B;RBX1;RBL2;CCNA2;RBL1;DBF4;TFDP1;TF	WikiPathway_2021_Human
Oncogene Induced Cellular Senescence	34/46	0.002976229	0.141666607	2.517445896	14.64422984	ATF2;GSK3B;CDKN1B;SUV39H1;PTEN;FOXO3;RPTOR;SPRED1;CCND1;CHEK2;MLST8;AKT1;MAPK1;MAP2K1;PDPK1;TSC2;BRAF;DHCR24;TSC1;MAPK14;CDK6;CDK4;GLB1;RHEB;SP1;CDK1;MDM2;NF1;SPRY2;ATM;H	Elsevier_Pathway_Collection
Apoptosis WP254	57/84	0.003980017	0.101490442	1.876832443	10.37225651	HRK;TRADD;IGF1R;BBC3;CASP9;IKBKB;CASP8;CASP6;MYC;CASP4;TNFSF10;CASP2;AKT1;BOK;DFFB;HELLS;BNIP3L;MAP2K4;APAF1;SCAF11;TRAF2;TRAF1;TNFRSF1B;TNFRSF1A;IRF3;TRAF3;IRF1;IRF2;BIRC5;IRF5;I	WikiPathway_2021_Human
Transcription Of E2F Targets Under Negative Control By P107 (RBL1) And P130 (RBL2) In Complex With HDAC1 R-HSA-1362300	14/16	0.004234454	0.082663901	6.214434158	33.95878133	LIN54;HDAC1;LIN9;CCNA2;RBL2;RBL1;TFDP1;RBBP4;TFDP2;CDK1;E2F1;MYBL2;E2F4;E2F5	Reactome_2022
ErbB signaling pathway WP673	61/91	0.004537863	0.106814314	1.807793276	9.753585395	GSK3B;CDKN1B;ARAF;PIK3CB;ELK1;CRKL;CCND1;MYC;AKT3;NCK2;AKT1;NCK1;MAP2K4;MAP2K1;MAP2K2;PRKCB;PDPK1;GAB1;NRG1;PRKCA;NRG2;BTC;PIK3CA;SOS1;RAF1;CRK;SOS2;CAMK2D;SRC;TGFA;PIK3R	WikiPathway_2021_Human
p53 activity regulation	77/118	0.00461004	0.105914076	1.670308708	8.985457131	RPL5;GSK3B;STEAP3;DYRK2;CD82;CSE1L;SETD7;PTEN;BBC3;YY1;CASP9;PIDD1;CCND3;RASSF1;CCND2;CASP8;CCND1;KAT5;SESN3;TRIM28;PPP2R4;PPP1R13L;CHEK2;SESN1;AKT1;EP300;KAT8;SKP2;CSNK1G3;P	BioPlanet_2019
Apoptosis Block in Melanoma Cell	29/39	0.005174167	0.176956521	2.576009838	13.56031379	PXN;PTEN;CASP9;NRAS;BCL2L11;MYC;RPS6KA1;AKT1;MAPK1;PLCG1;JAK2;JAK1;MCL1;MAP2K1;MAP2K2;APAF1;BAD;PDPK1;STAT1;STAT3;BRAF;MITF;PTK2;KIT;BCL2;MDM2;RAF1;TLN1;VCL	Elsevier_Pathway_Collection
ErbB signaling pathway	57/85	0.005891962	0.187364394	1.809609626	9.290836606	GSK3B;CDKN1B;ARAF;PIK3CB;ELK1;CRKL;MYC;AKT3;NCK2;AKT1;NCK1;MAP2K4;MAP2K1;MAP2K2;PRKCB;GAB1;NRG1;PRKCA;NRG2;BTC;PIK3CA;RAF1;SOS1;CRK;SOS2;CAMK2D;SRC;TGFA;PIK3R2;PIK3R1;CBL;E	KEGG_2021_Human
Cell Cycle, Mitotic R-HSA-69278	305/523	0.007538056	0.127777616	1.247826726	6.099116261	ANKLE2;NUP107;CCNH;CCP110;PPP2R2A;SMC3;SMC4;GOLGA2;PSMD9;PSMD6;CDC23;PSMD5;PPP2R1A;MYC;CDC26;AKT3;PSMD3;CDC27;AKT1;BTRC;SKP2;CDK5RAP2;TMPO;NUP214;NUP210;ESCO1;PRKCB;CS	Reactome_2022
Nicotinic acetylcholine receptor role in the regulation of apoptosis	15/18	0.007668956	0.133890954	4.438828531	21.61964619	CHRNB1;BAD;PDPK1;SRC;PIK3R1;FOXO3;PIK3CG;PTK2;CHRNG;PIK3CA;TERT;AKT1;PTK2B;AGRN;YWHAH	BioPlanet_2019
Apoptosis Block by Death Receptor Signaling in Cancer	36/51	0.007926267	0.205362383	2.132146914	10.31441644	FAF1;TRADD;XIAP;CASP9;IKBKB;MAPK9;MAPK8;CASP8;TNFSF10;CASP2;RIPK1;FADD;BID;MAP2K7;MAP3K7;MAP2K4;CASP8AP2;NGFR;APAF1;CRADD;DAPK1;TNFSF15;TNFRSF10B;TRAF2;CFLAR;NGF;NFKB1;TNFRS	Elsevier_Pathway_Collection
EGF receptor (ErbB1) signaling pathway	26/35	0.008197913	0.138246617	2.565685224	12.32523295	SRC;PIK3R2;PIK3CB;PIK3R1;WASL;EGFR;GNAI1;NRAS;PAK1;NCK2;MAPK1;PIP5K1C;PLCG1;NCK1;PTPN1;GSN;STAT1;STAT3;GAB1;PTPN11;PTK2;PIK3CA;RASA1;KRAS;SOS1;TLN1	BioPlanet_2019
Regulation Of TP53 Activity Thru Acetylation R-HSA-6804758	22/29	0.009916294	0.148958642	2.790778705	12.87546956	HDAC2;BRD1;MEAF6;HDAC1;BRPF3;CHD3;GATAD2B;PML;GATAD2A;ING5;ING3;PDZD2;RBBP4;KAT6A;AKT3;AKT1;EP300;PIP4K2B;PIP4K2C;RBBP7;BRD7;MTA2	Reactome_2022
Sonic Hedgehog (SHH) receptor PTCH1 regulation of cell cycle	11-Oct	0.009952693	0.149189866	8.875354108	40.9146022	CCNB1;CDK7;SHH;XPO1;CCNH;PTCH1;CDK1;MNAT1;CDC25A;CDC25B	BioPlanet_2019
H19 action Rb-E2F1 signaling and CDK-Beta-catenin activity WP3969	14-Dec	0.011703344	0.211345895	5.325651681	23.6878633	MED1;CDK8;JAG1;H2AFY;CCND1;CDK4;CDH1;E2F1;TULP3;PMAIP1;CTNNB1;SOX4	WikiPathway_2021_Human
Retinoblastoma gene in cancer WP2446	57/87	0.012086775	0.211345895	1.688608555	7.456293214	TOP2A;CDKN1B;MCM7;SUV39H1;FAF1;HMGB1;SMC3;SMC2;CCND3;CCND1;SIN3A;MYC;SKP2;RFC5;PRMT2;SMC1A;DCK;CDC25A;CDC25B;MSH6;CCNA2;TFDP1;TFDP2;RBP1;MCM3;MCM4;MCM6;RAF1;DNMT1;PRK	WikiPathway_2021_Human
DNA damage response (only ATM dependent) WP710	70/110	0.015445704	0.216114234	1.555555556	6.487326767	GSK3B;WNT2B;CDKN1B;IRS1;PTEN;PIK3C2G;PIK3CB;HMGB1;PIK3C2A;PIK3CG;PIK3C2B;BBC3;CCND3;CCND2;CCND1;PPP2R5E;MYC;AKT3;AKT1;RAC1;MAP3K7;MAP3K4;WNT5B;WNT5A;AXIN1;PPP2R5C;RHOA;RBI	WikiPathway_2021_Human
IFNG -> ARRB1/STAT1 Signaling	10-Sep	0.017236339	0.312105826	7.987064489	32.43335542	STAT5A;IFNGR1;STAT1;IFNGR2;STAT3;ARRB1;JAK2;PTPN2;JAK1	Elsevier_Pathway_Collection
Apoptosis modulation by HSP70	15/19	0.018567801	0.198931239	3.328767123	13.26955197	MAP3K1;APAF1;NFKB1;TNFRSF1A;CASP9;MAPK10;CASP8;AIFM1;CASP6;CASP2;FAS;RIPK1;FADD;BID;HSPA1B	BioPlanet_2019
Frizzled Receptors -> ARRB1/ARRB2 Canonical Signaling	13/16	0.019346911	0.312105826	3.846257989	15.17434363	GSK3B;CSNK1A1;AXIN1;LRP5;ARRB1;ARRB2;CSNK1E;LRP6;APC;DVL1;CTNNB1;TCF4;BTRC	Elsevier_Pathway_Collection
NOTCH -> RBPJ/HES/HEY Signaling	13/16	0.019346911	0.312105826	3.846257989	15.17434363	JAG1;CTBP1;HDAC1;MAML1;RFNG;RBPJ;DLL1;LFNG;NCOR2;ADAM17;HEY1;DVL1;NUMB	Elsevier_Pathway_Collection
Regulation Of PLK1 Activity At G2/M Transition R-HSA-2565942	56/87	0.020959293	0.241300582	1.605144647	6.204161968	CEP57;CEP164;CCP110;PPP1CB;ACTR1A;PCM1;PPP2R1A;CNTRL;CEP250;NEK2;BTRC;CEP290;NINL;CDK5RAP2;OFD1;HSP90AA1;BORA;PPP1R12A;FBXW11;HAUS3;CSNK1D;HAUS6;PRKCA;CSNK1E;HAUS5;TUBG1;	Reactome_2022
p75NTR Negatively Regulates Cell Cycle Via SC1 R-HSA-193670	6-Jun	0.022149467	0.248627772	56400	214880.719	NGFR;HDAC2;HDAC3;PRDM4;HDAC1;NGF	Reactome_2022
Cell cycle negative regulation by p75 neurotrophin receptor	6-Jun	0.022149467	0.210491535	56400	214880.719	NGFR;HDAC2;HDAC3;PRDM4;HDAC1;NGF	BioPlanet_2019
BH3-only Proteins Associate With And Inactivate Anti-Apoptotic BCL-2 Members R-HSA-111453	9-Aug	0.029607968	0.295421725	7.098942598	24.9862318	BCL2L11;BAD;STAT3;BCL2;PMAIP1;BMF;BID;BBC3	Reactome_2022
Activation of pro-caspase 8	9-Aug	0.029607968	0.246911325	7.098942598	24.9862318	CASP8;TRADD;TNFSF10;FAS;TNFRSF10B;RIPK1;TRAF2;FADD	BioPlanet_2019
Suppression of Caspase Activity in Cancer	31/46	0.034024371	0.391652639	1.835146813	6.204040831	APP;ROCK1;DIABLO;HTRA2;XIAP;HIF1A;CASP9;CASP8;AIFM1;CASP6;CASP4;BID;SPTAN1;DFFB;ENDOG;GSN;APAF1;PARP1;STAT3;MITF;CFLAR;NFKB1;ARHGEF3;GAS2;ACIN1;BIRC5;CTNNB1;BIRC6;XAF1;BIRC2;BIRC	Elsevier_Pathway_Collection
Cell cycle: G1/S checkpoint	20/28	0.037065426	0.291032233	2.219281664	7.312689898	GSK3B;CDKN2B;SMAD4;CDKN1B;SMAD3;TGFB1;HDAC1;TGFB3;CDC25A;DHFR;CDK6;TFDP1;CCND1;CDK4;ABL1;E2F1;CDK1;ATM;SKP2;ATR	BioPlanet_2019
Apoptosis Block by Inhibitor of Apoptosis Proteins (IAPs) in Cancer	15/20	0.038043401	0.425190957	2.662730279	8.704538847	DIABLO;STAT3;HTRA2;XIAP;MITF;HIF1A;NFKB1;CASP9;ARHGEF3;BIRC5;CTNNB1;BIRC6;XAF1;BIRC2;BIRC3	Elsevier_Pathway_Collection
GRB7 Events In ERBB2 Signaling R-HSA-1306955	5-May	0.041800806	0.377358839	47000	149217.4641	GRB7;ERBB3;ERBB2;NRG1;NRG2	Reactome_2022
GRB7 events in ERBB2 signaling	5-May	0.041800806	0.313294929	47000	149217.4641	GRB7;ERBB3;ERBB2;NRG1;NRG2	BioPlanet_2019
TLR -> ARRB2/NF-kB Signaling	5-May	0.041800806	0.453602764	47000	149217.4641	TRAF6;TAB2;ARRB2;TAB1;MAP3K7	Elsevier_Pathway_Collection
Apoptosis of Mantle Cell Deregulation	13/17	0.042442364	0.453602764	2.884386512	9.113531472	BAD;XIAP;CFLAR;CASP9;BCL2L11;CASP8;BCL2;MDM2;AKT1;BIRC5;FADD;BID;MCL1	Elsevier_Pathway_Collection
ESR1/ERBB Positive Luminal Breast Cancer	58/93	0.043012084	0.456836425	1.472125105	4.63170919	GSK3B;IRS1;PTEN;BRCA2;MYSM1;IGF1R;CASP9;MYC;AKT1;EP300;JAK2;JAK1;NCOA1;MAP2K4;MAP2K1;MAP2K2;APAF1;PARP1;PARP2;PDPK1;NCOA3;NRG1;PRKCA;SIRT1;MSH6;KAT2B;CREB1;MSH2;PIK3CA;MSH3	Elsevier_Pathway_Collection
Apoptosis Block by BCL2 Family Proteins in Cancer	27/40	0.04520332	0.471327295	1.843949392	5.709945574	HRK;FOXO3;HIF1A;BBC3;CASP9;MAPK9;MAPK8;BCL2L11;CASP8;AKT1;CASP2;PMAIP1;BMF;SFN;BID;BOK;MCL1;WWOX;APAF1;BAD;DAPK1;MITF;YWHAZ;NFKB1;MAPK10;BCL2;BCL2L2	Elsevier_Pathway_Collection

Suppl. Fig. 6. List of 49 hits term shown in figure 1s. Raw P.value and Adjusted P.value (Benjamini-Hochberg FDR correction, standard in EnrichR) along with Overlap: Number of input genes in the term / total genes in the term along with Odds.Ratio and Combined.Score (log odds * -log10(P)) for ranking strength are shown.

BL/6[C57] ECM Proteins

	3M	11M	14M	19M
Adgrg1	0	0	0	3.16E-06
Col1a1	0.40567	0.37471	0.38378	0.3678
Col28a1	0.00027	0.00019	0.00021	0.000
Col3a1	0.01216	0.01209	0.01312	0.012
Col4a2	0.00618	0.01476	0.01145	0.0231
Col4a3	0.00027	0.0001	0.00029	0.0001
Col4a4	4.13E-05	0.00014	0.00011	0.0001
Col5a1	0.00395	0.00295	0.00295	0.0036
Col5a3	0.01040	0.01259	0.01317	0.0236
Eln	0.00673	0.00684	0.00364	0.004
Emilin1	0.00112	0.00082	0.00066	0.0011
Fbn1	0.04548	0.05336	0.0535	0.0512
Fbn2	0.00396	0.00176	0.00183	0.0015
Lama1	4.86E-05	3.74E-05	3E-05	9.32E-05
Lama2	0.00015	0.00033	0.00034	0.0003
Tgfbi	0.0008	0.0014	0.00174	0.0018
Zan	0.00018	0.00010	0.00019	0.000
Col11a1	3.36E-06	2.18E-05	5.84E-05	4.64E-05
Col24a1	0.17605	0.19204	0.15430	0.1496
Col3afrag	0.0356	0.02466	0.04310	0.0460
Spp1	0	0	0	6.79E-06
Cd248	7.44E-05	3.39E-05	3.44E-05	2.38E-05
Col1a2	0.27769	0.28007	0.29727	0.2842
Col22a1	0.00027	7.94E-05	0.00012	5.35E-05
Col2a1	8.77E-05	9.74E-05	7.74E-05	0.0001
Col5a2	0.00631	0.00853	0.0076	0.009
Lama5	0.00104	0.00105	0.00132	0.0025
Col16a1	0.00016	0.00026	0.00020	0.0002
Col4avar2	0.0011	0.00426	0.00235	0.0041
Col4a6	1.24E-05	7.36E-05	4.89E-05	7.88E-05
Col8a1	0	2.3E-06	2.83E-07	0
Dmbt1	6.61E-05	0.00028	2.6E-05	2.01E-05
Fbln2	5.32E-05	5.42E-05	5.5E-05	9.52E-05
Lama3	0.0001	0.00012	0.00012	0.0005
Lamb1	0.00017	0.00027	0.00026	0.0005
Lamb2	0.00117	0.00150	0.00184	0.002
Lamb3	0.00014	0.00014	0.00011	0.0006
Lamc1	0.00083	0.00118	0.00131	0.0021
Mfap5	0.00052	0.00081	0.0008	0.0006
Nid1	0.00038	0.00056	0.00063	0.0008
Pxdn	1.18E-05	7.96E-06	7.19E-06	8.52E-05
Bgn	3.39E-05	0.0001	8.48E-05	0.000
Col15a1	9.14E-05	0.00011	0.00013	0.0006

Suppl. Fig. 7. Average proportional values of ECM proteins measured by proteomics analysis from BL6/[C57] mice at 3,11, 14, or 19 months of age. n=5 mice per time point.

BL/6^NZB ECM Proteins

Proteins	3M	11m	14m	19m
Adgrg1	0	9.17E-05	0.00064	0.0001
Col1a1	0.30380	0.28131	0.36833	0.2726
Col28a1	0.00038	0.00020	0.00040	0.0001
Col3a1	0.01026	0.00998	0.01126	0.0098
Col4a2	0.07372	0.06412	0.06146	0.0608
Col4a3	0.00174	0.00170	0.00167	0.0019
Col4a4	0	0.00011	0.00028	0.000
Col5a1	0.00678	0.00381	0.0057	0.0050
Col5a3	0.01830	0.03019	0.04581	0.0414
Eln	0.03142	0.00778	0.00813	0.0056
Emilin1	0.00633	0.0021	0.00215	0.001
Fbn1	0.12800	0.0697	0.10548	0.0935
Fbn2	0.01713	0.00904	0.00753	0.0057
Lama1	0.00015	0.00011	1.21E-05	0.0001
Lama2	0.00062	0.00051	0.00057	0.0008
Tgfbi	0.00103	0.0020	0.00224	0.0018
Zan	5.67E-06	6.31E-06	1.46E-05	5.45E-06
Col11a1	0	0	0	0
Col24a1	0	0	0	0
Col3aftag	0	0	0	0
Spp1	0	0	0	0
Cd248	0.00011	0.0002	0.00012	0.0001
Col1a2	0.37596	0.39902	0.34748	0.3772
Col22a1	0.00035	0.00049	0.00026	0.0004
Col2a1	9.72E-05	0.000	0.00012	0.000
Col5a2	0.00295	0.00590	0.00473	0.0061
Lama5	0.00359	0.00563	0.00346	0.0048
Col16a1	0.00031	0.00073	0.00016	0.0006
Col4aVar2	0.00391	0.02128	0.00861	0.0248
Col4a6	0.00021	0.00070	7.99E-05	0.0009
Col8a1	0	1.41E-05	3.37E-06	8.40E-06
Dmbt1	0.00095	0.00669	0.0017	0.0070
Fbln2	0.00011	0.00033	8.00E-05	0.0003
Lama3	0.00063	0.00227	0.00027	0.0016
Lamb1	0.00059	0.00173	0.00059	0.0015
Lamb2	0.0023	0.00619	0.0027	0.0062
Lamb3	0.00114	0.00278	0.00027	0.0020
Lamc1	0.00196	0.00444	0.00178	0.0043
Mfap5	0.00038	0.00082	0.00041	0.0012
Nid1	0.0028	0.00723	0.00193	0.0077
Pxdn	3.38E-05	0.00027	8.17E-05	0.0002
Bgn	6.85E-05	0.00654	0.00038	0.0065
Col15a1	0.00073	0.00594	0.00038	0.0061

Suppl. Fig. 8. Average proportional values of ECM proteins measured by proteomics analysis from BL6/^NZB mice at 3,11, 14, or 19 months of age. n=5 mice per time point.

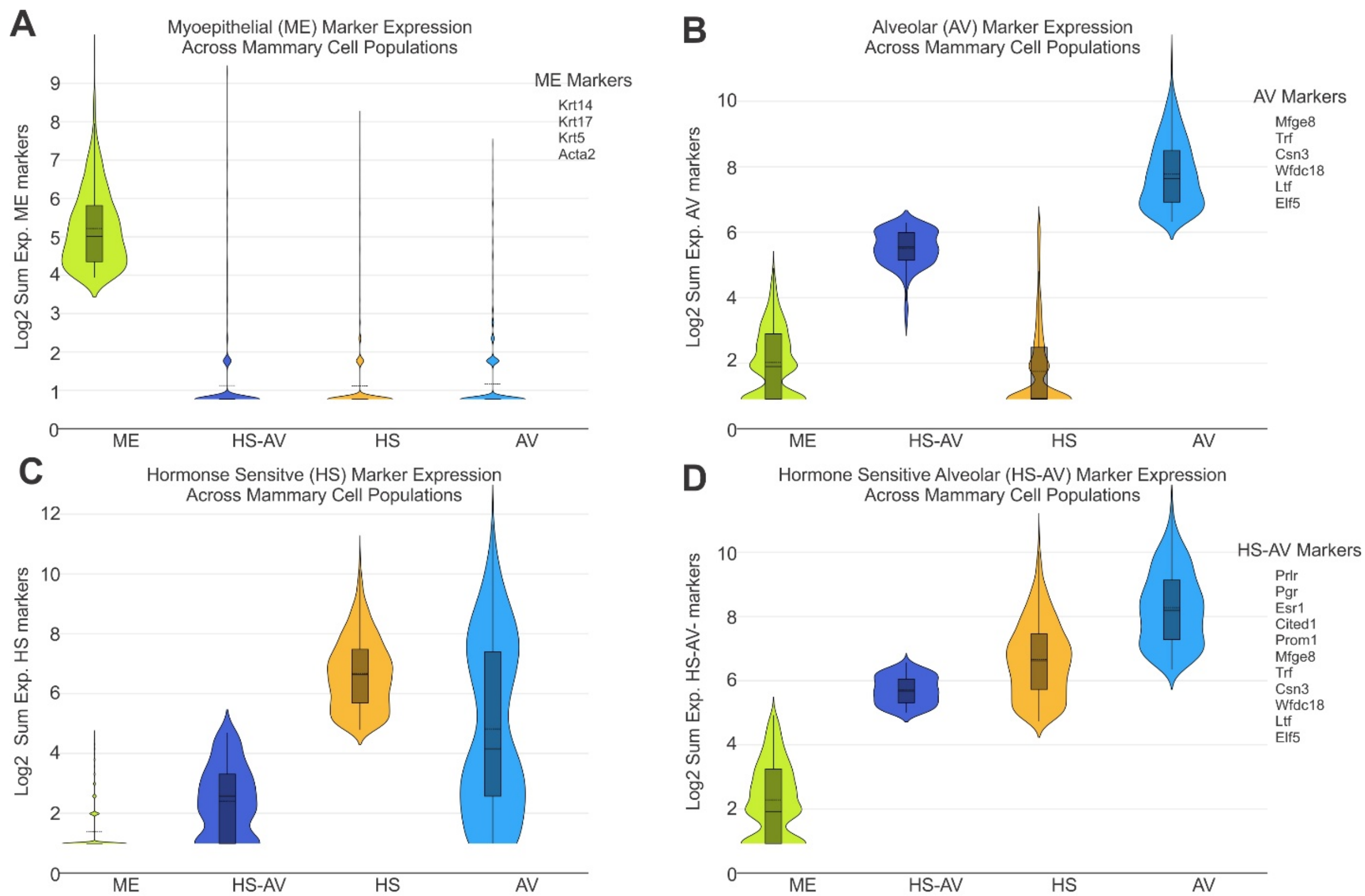

Suppl. Fig. 9. Violin plots of the Log2 Sum Expression values of the markers genes for myoepithelial (ME) (a), alveolar cells (AV) (b), hormone sensitive cells (HS) (c), or hormone sensitive alveolar cells (HS-AV) (d).

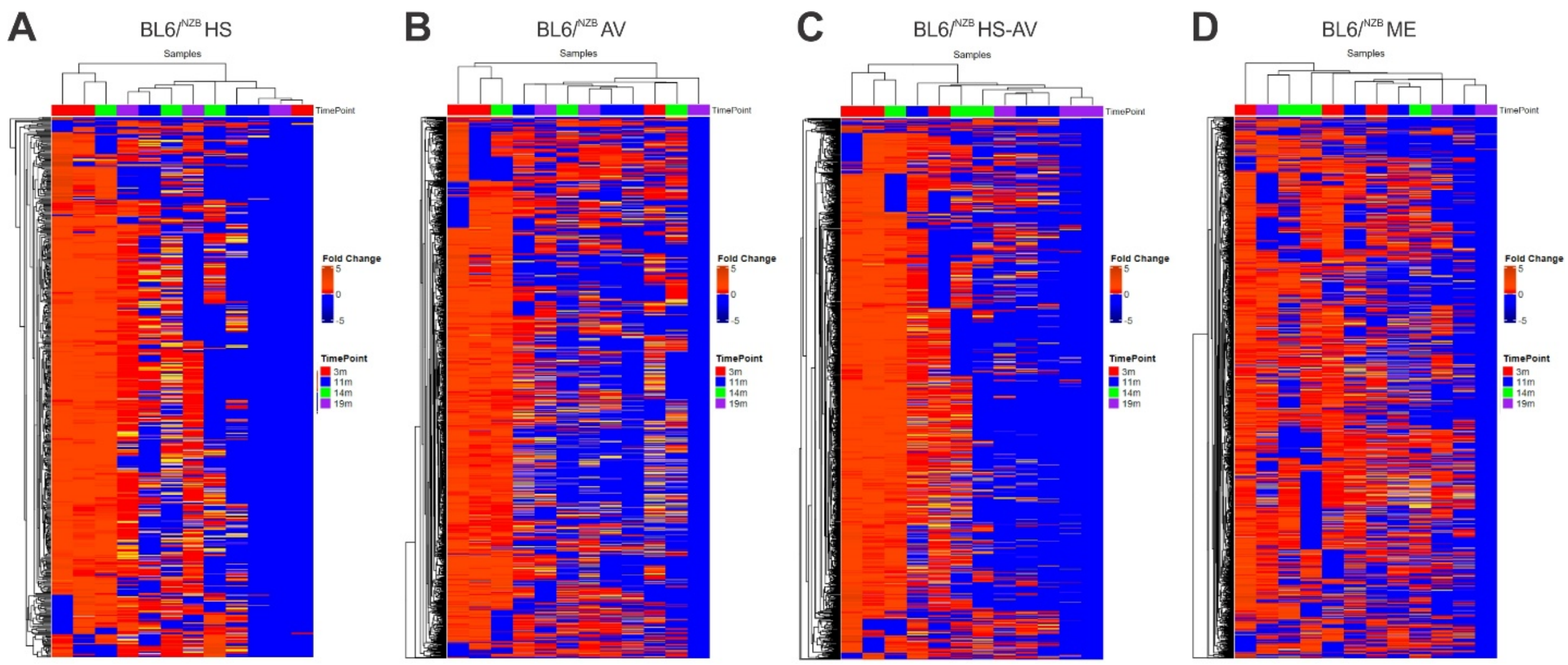

Suppl. Fig. 10. Gene expression heatmaps and hierarchical clustering of snRNAseq analysis of hormone sensitive (HS) cells (a), alveolar cells (AV) (b), hormone sensitive alveolar cells (HS-AV) (c), or Myoepithelial cells (ME) (d) from individual females (n=3 per age).

Bimodal genes in Hormone Sensitive Cells

Thsd7a	Cxcl15	2410006H16Rik	Cpne8	Pon2	Myl12a	Nras	Gng5
Phactr1	Tor1aip2	Tpd52l2	Cldn7	Rab27b	Cab39l	Eif3f	Stk32a
U2af1	Erp44	Cyba	Eif3h	Frmd4b	Slc38a1	Fermt1	Hmgb1
Tmem176a	Agtr1a	Sec61a1	Sipa1l3	Mast2	Ptpn1	Anxa6	Gm49980
Krt14	Arl6ip5	Gadd45g	Myl12b	Mindy2	Socs3	Smim14	Fgb
Dusp10	Ngf	Gfpt1	Gcnt2	Clic4	Aldoa	Plk2	Atxn7l3b
Alkbh5	Pgk1	Slc35f3	Dgkh	Gatb	Lamc2	Sem1	Sprr1a
Ctsr	Ido1	Ngly1	Pabpn1	Wnt5a	Itgb6	Uba52	Ftl1
Cystm1	Mapre1	Btg3	Celf4	Ctsb	Ecm1	Sec61g	Fn1
Rabac1	Tspan9	Nkain2	Tenm4	Gm10076	Rims2	Id3	

Suppl. Fig. 11. Bimodal (down at 3 m, up at 11 m, down at 14m, up at 19m) genes found in the hormone sensitive cell (HS) population. Red text indicates genes associated with c-MYC.

Bimodal genes in Alveolar Cells

Noct	Sh3pxd2b	Psmd8	Mycl	Palld	Pcp4	Krt15
Tomm6	Irf6	Syngr2	Eif5a	Swi5	Fgg	Aqp5
Ndufa11	Timm13	Gsto1	Cct8	Psmb4	Uqcrq	Fn1
Anapc13	Tmem86a	Itgb6	Psmb3	Chmp2a	Txnip	Il33
Acot1	Nfkbia	Cpne8	Tns4	Nop10	Urah	Mt2
Eif3b	Pa2g4	Nras	Fermt1	Ubl5	H2afj	
Msn	Itpkc	Fhl2	Map2k2	Set	Tgif1	
Arhgdia	Supt5	Prune2	Atp5g1	Krt14	Pdcd5	
Rnh1	Perp	Mob4	Mrpl52	1810037I17Rik	Ywhag	
Lgals7	Ptpn13	Rab11b	Fam107b	Gtf2h5	Dcn	

Suppl. Fig. 12. Bimodal (down at 3 m, up at 11 m, down at 14m, up at 19m) genes found in the alveolar cell (AV) population. Red text indicates genes associated with c-MYC.

Bimodal genes in Myoepithelial Cells

Gatb	Dusp10	Adgrg6	Lgals7	Il33
Ell2	Gsto1	Ier3	Slc2a3	Snrpg
Sprr1a	Macc1	Sh3pxd2b	45542	Gm10076
Sqstm1	Hadhb	Tmem86a	Aldh3a2	
Kalrn	Itpkc	Scmh1	Pon2	
Klf5	Creb5	Noct	Fn1	
Mcph1	R3hdml	Fermt1	Dnah11	
Myo1e	Fam107b	Csrp1	Itgb6	
Prune2	Mycl	Gm11808	Tns4	
4930523C07Rik	Fhl2	Krt15	Ctsr	

Suppl. Fig. 13. Bimodal (down at 3 m, up at 11 m, down at 14m, up at 19m) genes found in the Myoepithelial cell (ME) population. Red text indicates genes associated with c-MYC.

Bimodal genes in Hormone Sensitive Alveolar Cells

Ddb1	Aqp5	Enho	Uqcr10	Mt2	Tmem86a	Ywhag
Golph3	Arhgdia	Stt3a	Ier2	Pa2g4	Aoc1	Gpd2
Pdlim4	Manf	Nfkbia	Arpc1b	Egr1	Prune2	Phyh
Socs3	Greb1	Ifitm3	Bsdc1	Tgfb1	Ptpn13	Fn1
Dhx40	Laptm4a	Msn	Nras	Ubxn1	Fgb	Fhl2
Eif3b	Mycl	Rsl1d1	Mob4	Cpne8	Trir	Foxa1
Fam25c	R3hdml	Lman2	Rhob	Glod4	Eif4a1	Gnas
Sh3pxd2b	Gsto1	Ppp1r15a	Rpn2	U2af1	Tceal9	Fam107b
Lamc2	Slc6a6	Gm49980	Selenos	Muc13	Spcs2	Krt6a
Palld	Perp	Fermt1	Irf6	Dnajb9	Ssr1	CtsAqp5

Suppl. Fig. 14. Bimodal (down at 3 m, up at 11 m, down at 14m, up at 19m) genes found in the hormone sensitive alveolar cell (HS-AV) population. Red text indicates genes associated with c-MYC.

A

GSE analysis of cycling HS genes

term	pvalue	zscore	combined_scc	overlapping_genes	adjusted_pval	library	Category
Spliceosomal Assembly Homo sapiens h smPathway	0.031172	36.47253	126.4955846	U2AF1	0.111928012	BioCarta_2016	Altered Transcription
Polyadenylation of mRNA Homo sapiens h cpsfPathway	0.035	31.91186	106.9814138	PABPN1	0.111928012	BioCarta_2016	Altered Transcription
ATF-2 transcription factor network Homo sapiens c76c900a-618a-11e5-8ac5-06603eb7f303	0.022077	9.213822	35.13433	SOCS3,DUSP10	0.13045486	NCI-Nature_2016	Altered Transcription
KRAS Signaling Up	0.001177	6.835066	46.09876674	CAB39L,TMEM176A,TOR1AIP2,NGF,GADD45G	0.035321846	MSigDB_Hallmark_2020	Breast Cancer related
Epithelial Mesenchymal Transition	0.008134	5.367347	25.82614353	ECM1,WNT5A,FN1,LAMC2	0.122008477	MSigDB_Hallmark_2020	Breast Cancer related
TNF-alpha Signaling via NF-kB	0.044794	3.952177	12.27422205	BTG3,SOCS3,PLK2	0.154819445	MSigDB_Hallmark_2020	Breast Cancer related
Hypoxia	0.044794	3.952177	12.27422205	PGK1,GCNT2,ALDOA	0.154819445	MSigDB_Hallmark_2020	Breast Cancer related
p53 pathway Homo sapiens P00059	0.032118	7.472991	25.69473909	HMGB1,GADD45G	0.248407039	Panther_2016	Breast Cancer related
Metaplastic carcinoma of the breast	1.25E-06	9.610103	130.624419	NRAS,SEC61G,CLDN7,KRT14,ID3,LAMC2,U2AF1,HMGB1,IDO1	0.001242452	Rare_Diseases_AutoRIF_Gene_Lists	Breast Cancer related
Thyroid cancer Hurthle cell	0.007721	5.451606	26.5154226	ECM1,NRAS,KRT14,FN1	0.353747186	Rare_Diseases_AutoRIF_Gene_Lists	Breast Cancer related
Noonan syndrome KRAS	0.019984	9.736829	38.09833966	PTPN1,NRAS	0.444636307	Rare_Diseases_AutoRIF_Gene_Lists	Breast Cancer related
Skin cancer non melanoma childhood	0.022077	9.213822	35.13433	NRAS,HMGB1	0.460863662	Rare_Diseases_AutoRIF_Gene_Lists	Breast Cancer related
Collagen-Containing Extracellular Matrix (GO:0062023)	0.000793	5.053746	36.07914484	FGB,ECM1,WNT5A,ANXA6,FN1,LAMC2,CTSB	0.041260636	GO_Cellular_Component_2025	ECM
Endocytic Vesicle Membrane (GO:0030666)	0.027624	4.814571	17.27977216	WNT5A,CYBA,UBA52	0.208556829	GO_Cellular_Component_2025	ECM
Extracellular exosome	3.14E-06	3.293374	41.73416341	5,ARL6IP5,ANXA6,PGK1,THSD7A,ITGB6,CTSB,FGB,SLC38A1,WNT5A,GFPT1,FN1,RAB27B,ERP44,KRT14,EIF3H,CYSTM1,ALDOA,UBA52	0.000651841	Jensen_COMPARTMENTS	ECM
Extracellular vesicle	3.46E-06	3.2738	41.16416359	5,ARL6IP5,ANXA6,PGK1,THSD7A,ITGB6,CTSB,FGB,SLC38A1,WNT5A,GFPT1,FN1,RAB27B,ERP44,KRT14,EIF3H,CYSTM1,ALDOA,UBA52	0.000651841	Jensen_COMPARTMENTS	ECM
Extracellular organelle	3.49E-06	3.27241	41.12378499	CPNE8,CLIC4,ECM1,CAB39L,MYL12A,MYL12B,RIMS2,NRAS,GNG5,ARL6IP5,ANXA6,PGK1,THSD7A,ITGB6,CTSB,FGB,SLC38A1,WNT5A,GFPT1,FN1,RAB27B,ERP44,KRT14,EIF3H,CYSTM1,ALDOA,UB	0.000651841	Jensen_COMPARTMENTS	ECM
membrane-bounded vesicle	1.36E-05	2.886473	32.33908107	CPNE8,CLIC4,ECM1,CAB39L,MYL12A,MYL12B,RIMS2,NRAS,GNG5,ARL6IP5,ANXA6,PGK1,THSD7A,ITGB6,RABAC1,CTSB,FGB,SLC38A1,WNT5A,GFPT1,FN1,CYBA,RAB27B,NGF,ERP44,KRT14,EIF3H,CYSTM1,ALDOA,UBA52	0.001910787	Jensen_COMPARTMENTS	ECM
Beta3 integrin cell surface interactions Homo sapiens c2800165-618d-11e5-8ac5-06603eb7f303	0.000653	19.61941	143.8796135	FGB,FN1,HMGB1	0.042461917	NCI-Nature_2016	ECM
Beta5 beta6 beta7 and beta8 integrin cell surface interactions Homo sapiens 108637d6-618e-11e5-8ac5-06603eb7f303	0.002016	34.46926	213.9372418	FN1,ITGB6	0.049173319	NCI-Nature_2016	ECM
Beta1 integrin cell surface interactions Homo sapiens 2fd0bc63-618d-11e5-8ac5-06603eb7f303	0.00227	12.44236	75.75129075	FGB,FN1,LAMC2	0.049173319	NCI-Nature_2016	ECM
Integrin signalling pathway Homo sapiens P00034	0.023913	5.100103	19.04049001	NRAS,FN1,ITGB6	0.248407039	Panther_2016	ECM
Glycolysis Homo sapiens P00024	0.002016	34.46926	213.9372418	PGK1,ALDOA	0.064514001	Panther_2016	Energy Production
N-acetylglucosamine metabolism Homo sapiens P02756	0.02347	51.06667	191.6039295	GFPT1	0.248407039	Panther_2016	Energy Production

B

GSE analysis of non-cycling HS genes

term	pvalue	zscore	combined_score	overlapping_genes	adjusted_pvalue	library	Category
HIF-1-alpha transcription factor network Homo sapiens 20ef2b81-6193-11e5-8ac5-06603eb7f303	2.65E-08	5.354693	93.42037	JUN,NPM1,EGLN3,SMAD4,PFKFB3,SMAD3,CITED2,BHLHE41,ARNT,FURIN,ENO1,ETS1,NDRG1,HIF1A,HK2,LDHA,CREB1,SP1,ID2,BHLHE40,PLIN2	1.75E-06	NCI-Nature_2016	Altered Transcription
Validated targets of C-MYC transcriptional activation Homo sapiens 61d3b115-6196-11e5-8ac5-06603eb7f303	3.83E-08	4.715751	80.54035	EIF4A1,HSP90AA1,NPM1,SMAD4,SMAD3,MAX,HSPA4,RPL11,ODC1,NME2,ENO1,MTDH,LDHA,PDCD10,NCL,ID2,UBTF,PIM1,TAF4B,PTMA,GAPDH,EIF4E,EIF4G1	1.89E-06	NCI-Nature_2016	Altered Transcription
TNF-alpha Signaling via NF-kB	1.23E-17	4.654092	181.206	TNFAIP8,B4GALT1,IRS2,SLC2A3,LITAF,AREG,ETS2,IFIH1,CCND1,NAMPT,PDE4B,DENND5A,PHLDA1,IER3,MAP2K3,KDM6B,TGIF1,TSC22D1,DUSP1,PLAUR,F3,RHOB,IRF1,TRIB1,PLPP3,SGK1,BIRC2,BIRC3,PPP1R15A,PFKFB3,RELA,DNAJB4,HES1,EGR1,JUN,EGR2,EGR3,SMAD3,GADD45B,G0S2,SOD2,PNRC1,KLF2,FOSL2,EIF1,NR4A2,PER1,NR4A1,RCAN1,NR4A3,ID2,MAFF,BHLHE40,BCL3,KLF9,REL,NFE2L2	3.08E-16	MSigDB_Hallmark_2020	Breast Cancer related
Insulin/IGF pathway-mitogen activated protein kinase kinase/MAP kinase cascade Homo sapiens P00032	5.62E-05	6.006346	58.78452	MAP2K3,MAP2K2,RPS6KB1,INSR,RASA1,IRS2,RAF1,SOS2,IGF2R,IGF1R	0.001854	Panther_2016	Energy Production

Suppl. Fig. 15. Gene set enrichment analysis of the bimodal (a) or non-bimodal (b) genes found in the hormone sensitive (HS) population.

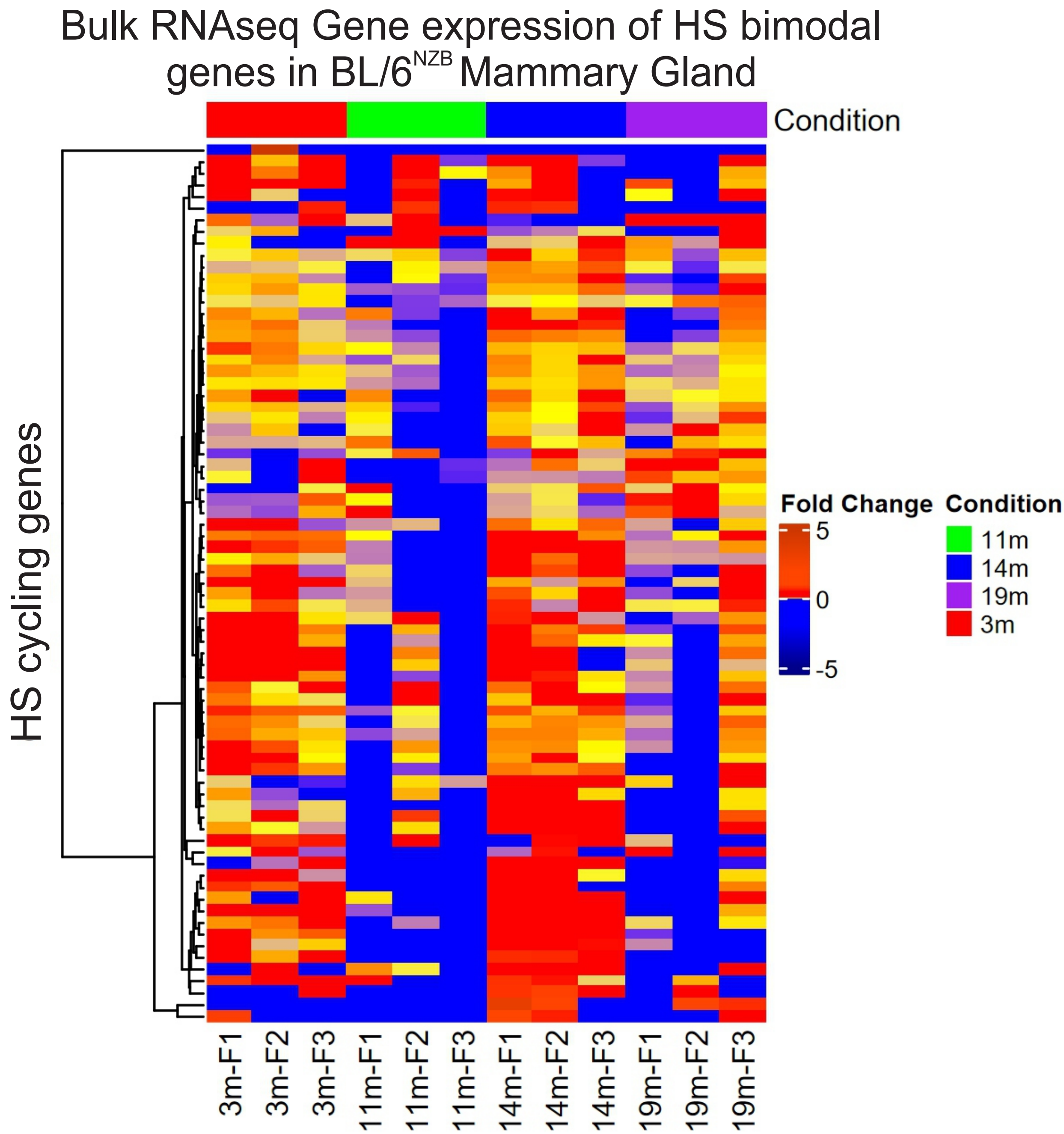

Suppl. Fig. 16. Heatmap of the HS bimodal genes signature in the bulk RNAseq data from BL/6^{NZB} mice at 3,11, 14, or 19 months of age. N=3 mice per time point.

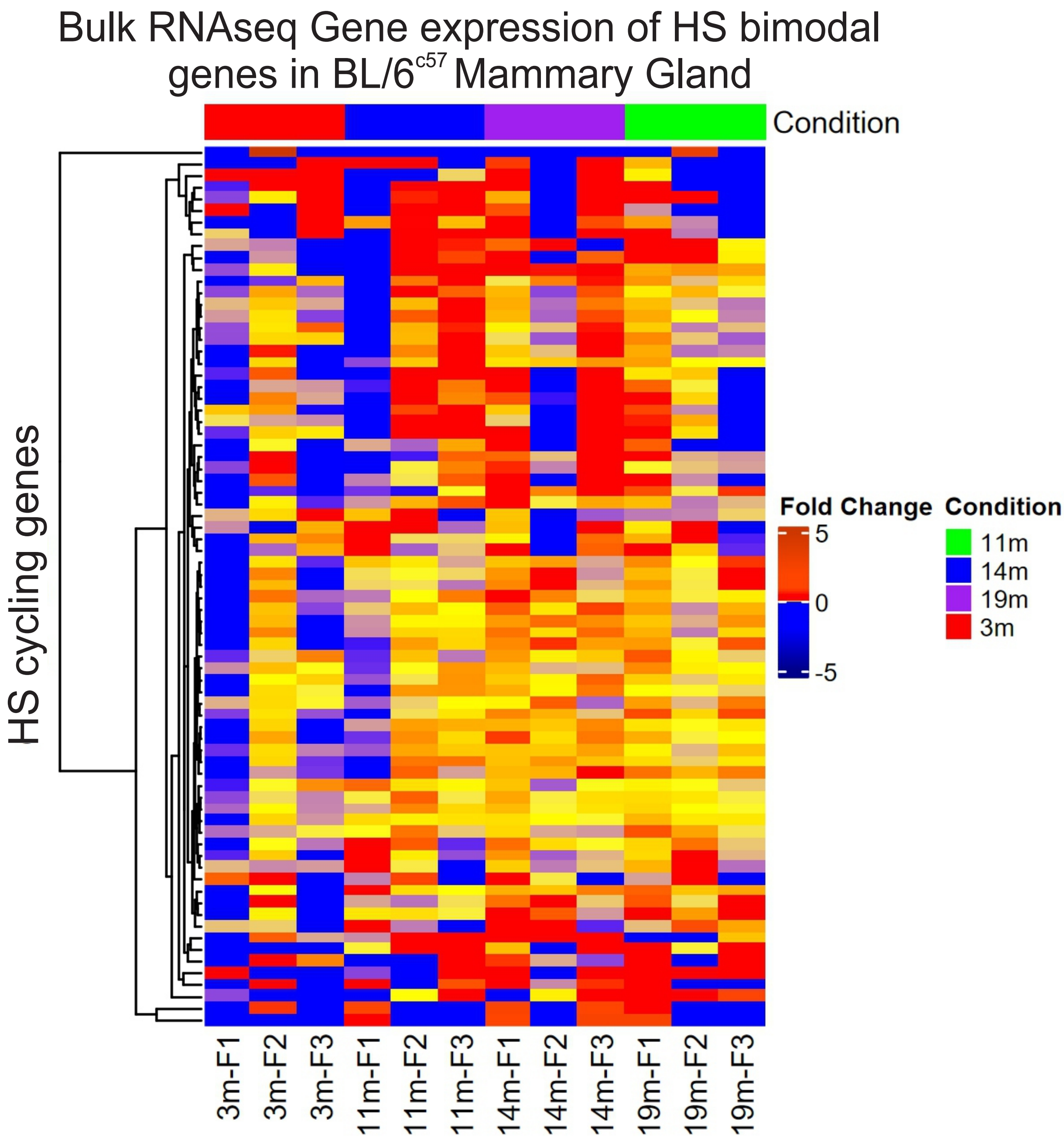

Suppl. Fig. 17. Heatmap of the HS bimodal genes signature in the bulk RNAseq data from BL/6^{C57} mice at 3,11, 14, or 19 months of age. N=3 mice per time point.

GSE analysis of bimodal AV genes

term	pvalue	zscore	combined_score	overlapping_genes	adjusted_pvalue	library	Category
Regulation of Splicing through Sam68 Homo sapiens h sam68Pathway	0.032036	34.59375	119.0338	MAP2K2	0.11569004	BioCarta_2016	Altered Transcription
Transcription Factor TFIIH Core Complex (GO:0000439)	0.028878	38.91992	137.9583	GTF2H5	0.19130834	GO_Cellular_Component_2025	Altered Transcription
Transcription Factor TFIIH Holo Complex (GO:0005675)	0.035183	31.13281	104.2074	GTF2H5	0.19130834	GO_Cellular_Component_2025	Altered Transcription
Validated transcriptional targets of deltaNp63 isoforms Homo sapiens 7d7360a8-6196-11e5-8ac5-06603eb7f303	0.00941	14.68586	68.52324	PERP,KRT14	0.05207126	NCI-Nature_2016	Altered Transcription
TNFR2 Signaling Pathway Homo sapiens h tnfr2Pathway	0.038321	28.30114	92.3118	NFKBIA	0.11569004	BioCarta_2016	Breast Cancer related
Erythropoietin mediated neuroprotection through NF-kB Homo sapiens h eponfkbPathway	0.041448	25.94141	82.57978	NFKBIA	0.11569004	BioCarta_2016	Breast Cancer related
Epithelial-myoepithelial carcinoma	0.002767	11.57326	68.16836	NFKBIA,NRAS,KRT14	0.16295211	Rare_Diseases_AutoRIF_Gene_Lists	Breast Cancer related
Thyroid cancer Hurthle cell	0.003869	6.707551	37.2581	NRAS,KRT14,FN1,MSN	0.16295211	Rare_Diseases_AutoRIF_Gene_Lists	Breast Cancer related
Noonan syndrome KRAS	0.013825	11.90895	50.98534	NRAS,MAP2K2	0.22970987	Rare_Diseases_AutoRIF_Gene_Lists	Breast Cancer related
Skin cancer non melanoma childhood	0.015294	11.26927	47.10909	NRAS,DCN	0.22970987	Rare_Diseases_AutoRIF_Gene_Lists	Breast Cancer related
Intracellular Membraneless Organelle (GO:0043232)	0.025184	2.301883	8.47445	MYCL,SET,ARHGDIA,KRT15,SH3PXD2B,KRT14,MSN,PA2G4,GTF2H5	0.19130834	GO_Cellular_Component_2025	ECM
Extracellular exosome	6.58E-09	4.805908	90.54086	CPNE8,RNH1,PDCD5,AQP5,PSMD8,NRAS,PSMB4,SYNGR2,H2AFJ,PSMB3,ARHGDIA,ITGB6,CCT8,LGALS7,YWHAG,EIF5A,GSTO1,FGG,FN1,MSN,PA2G4,PTPN13,RAB11B,KRT15,KRT14,CHMP2A,IRF6,EIF3B	1.52E-06	Jensen_COMPARTMENTS	ECM
Extracellular vesicle	7.38E-09	4.777339	89.45417	CPNE8,RNH1,PDCD5,AQP5,PSMD8,NRAS,PSMB4,SYNGR2,H2AFJ,PSMB3,ARHGDIA,ITGB6,CCT8,LGALS7,YWHAG,EIF5A,GSTO1,FGG,FN1,MSN,PA2G4,PTPN13,RAB11B,KRT15,KRT14,CHMP2A,IRF6,EIF3B	1.52E-06	Jensen_COMPARTMENTS	ECM
Extracellular organelle	7.44E-09	4.77531	89.37715	CPNE8,RNH1,PDCD5,AQP5,PSMD8,NRAS,PSMB4,SYNGR2,H2AFJ,PSMB3,ARHGDIA,ITGB6,CCT8,LGALS7,YWHAG,EIF5A,GSTO1,FGG,FN1,MSN,PA2G4,PTPN13,RAB11B,KRT15,KRT14,CHMP2A,IRF6,EIF3B	1.52E-06	Jensen_COMPARTMENTS	ECM
membrane-bounded vesicle	3.85E-07	3.799764	56.12721	CPNE8,RNH1,PDCD5,AQP5,PSMD8,NRAS,PSMB4,SYNGR2,H2AFJ,PSMB3,ARHGDIA,ITGB6,CCT8,LGALS7,YWHAG,EIF5A,IL33,GSTO1,FGG,FN1,MSN,PA2G4,PTPN13,RAB11B,KRT15,KRT14,CHMP2A,IRF6,EIF3B	5.60E-05	Jensen_COMPARTMENTS	ECM
Beta5 beta6 beta7 and beta8 integrin cell surface interactions Homo sapiens 108637d6-618e-11e5-8ac5-06603eb7f303	0.001371	42.15873	277.9317	FN1,ITGB6	0.03056502	NCI-Nature_2016	ECM
Beta3 integrin cell surface interactions Homo sapiens c2800165-618d-11e5-8ac5-06603eb7f303	0.008619	15.40379	73.22579	FGG,FN1	0.05207126	NCI-Nature_2016	ECM
Beta1 integrin cell surface interactions Homo sapiens 2fd0bc63-618d-11e5-8ac5-06603eb7f303	0.019519	9.856647	38.79924	FGG,FN1	0.08100501	NCI-Nature_2016	ECM
Integrin signalling pathway Homo sapiens P00034	0.001666	8.534513	54.59636	NRAS,MAP2K2,FN1,ITGB6	0.0150151	Panther_2016	ECM

Suppl. Fig. 18. Gene set enrichment analysis of the bimodal genes found in the alveolar cell (AV) population.

GSE analysis of bimodal HS-AV genes

term	pvalue	zscore	combined_score	overlapping_genes	adjusted_pvalue	library	Category
Spliceosomal Assembly Homo sapiens h smPathway	0.027663994	41.2484472	147.9839017	U2AF1	0.12872992	BioCarta_2016	Altered Transcription
AP-1 transcription factor network Homo sapiens 3ce2f9c5-6189-11e5-8ac5-06603eb7f303	0.024363702	8.71949078	32.3899512	EGR1,TGFB1	0.114397214	NCI-Nature_2016	Altered Transcription
TNFR2 Signaling Pathway Homo sapiens h tnfr2Pathway	0.041211758	26.2437418	83.6921237	NFKBIA	0.12872992	BioCarta_2016	Breast Cancer related
Erythropoietin mediated neuroprotection through NF-kB Homo sapiens h eponfkbPathway	0.044569523	24.0555556	74.8297370	NFKBIA	0.12872992	BioCarta_2016	Breast Cancer related
TNF-alpha Signaling via NF-kB	7.14E-05	9.53737113	91.0494307	PPP1R15A,NFKBIA,SOCS3,EGR1,IER2,RHOB	0.00271487	MSigDB_Hallmark_2020	Breast Cancer related
Epithelial Mesenchymal Transition	0.000679747	7.78500986	56.7822276	TGFB1,FN1,LAMC2,PDLIM4,RHOB	0.00645759	MSigDB_Hallmark_2020	Breast Cancer related
p53 Pathway	0.033070333	4.48511251	15.2902808	PPP1R15A,TGFB1,PERP	0.125667264	MSigDB_Hallmark_2020	Breast Cancer related
Metaplastic carcinoma of the breast	4.60E-05	8.24528302	82.350120	FOXA1,NRAS,TGFB1,GNAS,LAMC2,U2AF1,KRT6A	0.01417684	Rare_Diseases_AutoRIF_Gene_Lists	Breast Cancer related
Urachal adenocarcinoma	0.00343887	25.4565217	144.404980	NRAS,GNAS	0.13178006	Rare_Diseases_AutoRIF_Gene_Lists	Breast Cancer related
Nonmedullary thyroid carcinoma with or without cell oxyphilia	0.005209724	9.15510078	48.1304565	NRAS,TGFB1,FN1	0.13536099	Rare_Diseases_AutoRIF_Gene_Lists	Breast Cancer related
Intracellular Membraneless Organelle (GO:0043232)	0.038431229	2.11263719	6.88484141	DDB1,MYCL,RSL1D1,ARHGDIA,SH3PXD2B,DNAJB9,MSN,PA2G4,PDLIM4	0.21897862	GO_Cellular_Component_2025	ECM
Collagen-Containing Extracellular Matrix (GO:0062023)	0.045253742	3.12640921	9.67770566	FGB,TGFB1,FN1,LAMC2	0.21897862	GO_Cellular_Component_2025	ECM
Extracellular exosome	7.77E-07	3.74837543	52.7308017	IFITM3,CPNE8,EIF4A1,ARPC1B,AQP5,UQCR10,NRAS,ARHGDIA,LMAN2,MUC13,DNAJB9,YWHAG,KRT6A,FGB,GSTO1,GREB1,FN1,MSN,PA2G4,PTPN13,RHOB,DDB1,GLOD4,GNAS,IRF6,EIF3B	0.00018008	Jensen_COMPARTMENTS	ECM
Extracellular vesicle	8.58E-07	3.72610637	52.0473909	IFITM3,CPNE8,EIF4A1,ARPC1B,AQP5,UQCR10,NRAS,ARHGDIA,LMAN2,MUC13,DNAJB9,YWHAG,KRT6A,FGB,GSTO1,GREB1,FN1,MSN,PA2G4,PTPN13,RHOB,DDB1,GLOD4,GNAS,IRF6,EIF3B	0.00018008	Jensen_COMPARTMENTS	ECM
Extracellular organelle	8.64E-07	3.72452447	51.9989674	IFITM3,CPNE8,EIF4A1,ARPC1B,AQP5,UQCR10,NRAS,ARHGDIA,LMAN2,MUC13,DNAJB9,YWHAG,KRT6A,FGB,GSTO1,GREB1,FN1,MSN,PA2G4,PTPN13,RHOB,DDB1,GLOD4,GNAS,IRF6,EIF3B	0.00018008	Jensen_COMPARTMENTS	ECM
Extracellular region part	1.02E-05	3.06491447	35.2385760	IFITM3,CPNE8,EIF4A1,ARPC1B,LAMC2,AQP5,UQCR10,NRAS,ARHGDIA,LMAN2,MUC13,DNAJB9,YWHAG,KRT6A,FGB,TGFB1,GSTO1,GREB1,FN1,MSN,PA2G4,PTPN13,RHOB,MANF,DDB1,GLOD4,GNAS,IRF6,EIF3B	0.00158693	Jensen_COMPARTMENTS	ECM
Beta1 integrin cell surface interactions Homo sapiens 2fd0bc63-618d-11e5-8ac5-06603eb7f303	0.001604525	14.1201137	90.8619048	FGB,FN1,LAMC2	0.02917507	NCI-Nature_2016	ECM
Beta3 integrin cell surface interactions Homo sapiens c2800165-618d-11e5-8ac5-06603eb7f303	0.009939917	14.2675753	65.7905944	FGB,FN1	0.07951933	NCI-Nature_2016	ECM
a6b1 and a6b4 Integrin signaling Homo sapiens 73d1a893-6186-11e5-8ac5-06603eb7f303	0.010848748	13.6025992	61.5341540	LAMC2,YWHAG	0.08011383	NCI-Nature_2016	ECM
Integrin signalling pathway Homo sapiens P00034	0.002189839	7.88596491	48.2930754	NRAS,ARPC1B,FN1,RHOB	0.05912565	Panther_2016	ECM
Pilomatrixoma	0.005355889	9.06122449	47.3862045	NRAS,TGFB1,FHL2	0.13536099	Rare_Diseases_AutoRIF_Gene_Lists	ECM

Suppl. Fig. 19. Gene set enrichment analysis of the bimodal genes found in the hormone sensitive alveolar cell (HS-AV) population.

GSE analysis of bimodal ME genes

term	pvalue	zscore	combined_score	overlapping_genes	adjusted_pvalue	library	Category
Hypoxia and p53 in the Cardiovascular system Homo sapiens h p53hypoxiaPathway	0.04421378	23.7345238	74.02130556	FHL2	0.044213779	BioCarta_2016	Breast Cancer related
TNF-alpha Signaling via NF-kB	0.00906188	7.52284264	35.38503216	SLC2A3,SQSTM1,IER3	0.07551569	MSigDB_Hallmark_2020	Breast Cancer related
Hypoxia	0.00906188	7.52284264	35.38503216	NOCT,SLC2A3,IER3	0.07551569	MSigDB_Hallmark_2020	Breast Cancer related
Hypopharyngeal cancer	0.03835418	6.8069392	22.19669261	LGALS7,SQSTM1	0.383541762	Rare_Diseases_AutoRIF_Gene_Lists	Breast Cancer related
Supraglottic laryngeal cancer	0.04279685	6.39832014	20.16296703	KLF5,SLC2A3	0.395268339	Rare_Diseases_AutoRIF_Gene_Lists	Breast Cancer related
Extracellular exosome	0.00386973	2.73792935	15.20802303	GSTO1,FN1,SLC2A3,KALRN,HADHB,ALDH3A2,MYO1E,CSRP1, KRT15,ITGB6,LGALS7,SQSTM1,CREB5	0.427018024	Jensen_COMPARTMENTS	ECM
Extracellular vesicle	0.00404679	2.72173173	14.99628095	GSTO1,FN1,SLC2A3,KALRN,HADHB,ALDH3A2,MYO1E,CSRP1, KRT15,ITGB6,LGALS7,SQSTM1,CREB5	0.427018024	Jensen_COMPARTMENTS	ECM
Extracellular organelle	0.00405969	2.72058108	14.98128315	GSTO1,FN1,SLC2A3,KALRN,HADHB,ALDH3A2,MYO1E,CSRP1, KRT15,ITGB6,LGALS7,SQSTM1,CREB5	0.427018024	Jensen_COMPARTMENTS	ECM
membrane-bounded vesicle	0.0126304	2.26835897	9.916469204	IL33,GSTO1,FN1,SLC2A3,KALRN,HADHB,ALDH3A2,MYO1E,CSRP1,KRT15,ITGB6,LGALS7,SQSTM1,CREB5	0.427018024	Jensen_COMPARTMENTS	ECM
Beta5 beta6 beta7 and beta8 integrin cell surface interactions Homo sapiens 108637d6-618e-11e5-8ac5-06603eb7f303	0.0006016	64.8520325	480.9377475	FN1,ITGB6	0.013836713	NCI-Nature_2016	ECM
Integrin signalling pathway Homo sapiens P00034	0.04431889	6.27272727	19.54797791	FN1,ITGB6	0.166513584	Panther_2016	ECM
Glomerulopathy with fibronectin deposits 2	0.02969228	36.5274725	128.4623071	FN1	0.350908758	Rare_Diseases_AutoRIF_Gene_Lists	ECM

Suppl. Fig. 20. Gene set enrichment analysis of the bimodal genes found in the myoepithelial cell (ME) population.

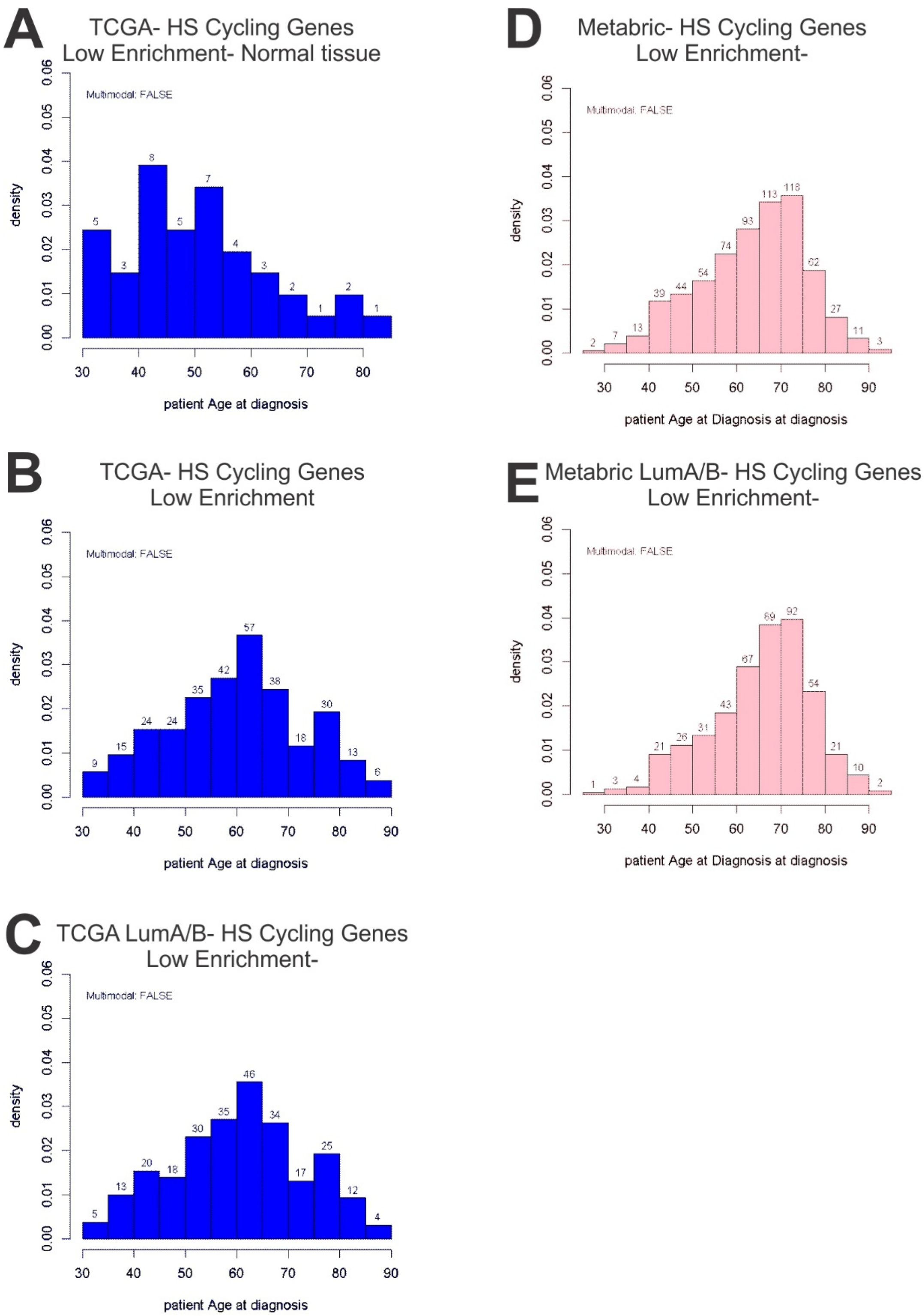

Suppl. Fig. 21. a) Histogram of patient age at diagnosis for TCGA patients not enriched (bottom two tertiles) for the HS bimodal gene signature in normal tissue. b) Histogram of

patient age at diagnosis for TCGA patients not enriched (bottom tertile) for the HS bimodal gene signature. c) Histogram of patient age at diagnosis for Lum A/B TCGA patients not enriched (bottom tertile) for the HS bimodal gene signature. d) Histogram of patient age at diagnosis for METABRIC patients not enriched (bottom tertile) for the HS bimodal gene signature. e) Histogram of patient age at diagnosis for Lum A/B METABRIC patients not enriched (bottom tertile) for the HS bimodal gene signature. Multimodality was assessed using a custom density-based test, where a kernel density estimate (bandwidth = 'SJ') identified significant peaks (local maxima) exceeding a height threshold of 75% of the maximum density, indicating multimodality if multiple peaks were detected. Density values are shown, with counts labeled above each bin.

n tot= 933	Bottom 2 tertiles	Top tertile	P VALUE	MISSING DATA
	N= 622	N= 311		
**ER STATUS**				
ER negative	117 (23%)	55 (21%)		
ER positive	387 (77%)	206 (79%)	n.s.	168 (18%)
**HER2 STATUS**				
HER2 negative	417 (85%)	220 (86%)		
HER2 positive	76 (15%)	37 (14%)	n.s.	183 (20%)
**METASTASIS**				
Mets negative	499 (98%)	256 (98%)		
Mets positive	10 (2%)	4 (2%)	n.s.	164 (18%)
**Node**				
Node negative	246 (48%)	134 (51%)		
Node positive	268 (52%)	127 (49%)		158 (17%)
**PR STATUS**				
PR NEGATIVE	166 (33%)	82 (32%)		
PR POSITIVE	336 (67%)	178 (68%)	n.s.	171 (18%)
**MENOPAUSE**				
PERI	19 (3%)	13 (5%)	n.s.	
POST	372 (68%)	201 (73%)	n.s.	
PRE	159 (29%)	61 (22%)	0.0449 vs rest	108 (12%)
**AGE**				
< 40	56 (9%)	41 (13%)	0.053 vs rest	
41-64	371 (60%)	191 (61%)	n.s.	
ABOVE 65	195 (31%)	79 (25%)	n.s.	0 (0%)
< or equal to 43	83 (13%)	61 (20%)	0.016 vs rest	
44-51	109 (18%)	67 (22%)	n.s.	
52-57	97 (16%)	41 (13%)	n.s.	
58-64	138 (22%)	63 (20%)	n.s.	
> 65	195 (31%)	79 (25%)	n.s.	0 (0%)
**Cancer subtypes**				
Normal	41 (7%)	72 (24%)	0.0001 vs rest	
Luminal A	287 (47%)	118 (39%)	n.s.	
Luminal B	169 (28%)	17 (6%)	0.0001 vs rest	
Basal	66 (11%)	70 (23%)	0.0001 vs rest	
Her2	41 (7%)	25 (8%)	n.s.	27 (3%)
**BRCA germline mutation**				
BRCA1	8 (1.4%)	4 (2%)	n.s.	
BRCA2	8 (1.4%)	7 (3%)	n.s.	
Both	16 (3%)	11 (5%)	n.s.	116 (13%)

Suppl. Fig. 22. Patient clinical information in the TCGA dataset with respect to the HS bimodal gene signature.
